# Integrative network analysis of circular RNAs reveals regulatory mechanisms for hepatic specification of human iPSC-derived endoderm

**DOI:** 10.1186/s13287-022-03160-z

**Published:** 2022-09-08

**Authors:** Fang Bai, Jinliang Duan, Daopeng Yang, Xingqiang Lai, Xiaofeng Zhu, Xiaoshun He, Anbin Hu

**Affiliations:** 1 Organ Transplant Center, The First Affiliated Hospital, Sun Yat-sen University, Guangdong Provincial Key Laboratory of Organ Donation and Transplant Immunology, Guangdong Provincial International Cooperation Base of Science and Technology (Organ Transplantation), Guangzhou, Guangdong China; 2 Zhongshan School of Medicine, Sun Yat-sen University, Guangzhou, Guangdong China; 3 Department of Cardiology, The Eighth Affiliated Hospital, Sun Yat-sen University, Shenzhen, Guangdong China

**Keywords:** Circular RNA, Human-induced pluripotent stem cell, ceRNA network, Hepatic specification, hsa_circ_004658

## Abstract

**Background:**

Human-induced pluripotent stem cell (hiPSC)-derived functional hepatic endoderm (HE) is supposed to be an alternative option for replacement therapy for end-stage liver disease. However, the high heterogeneity of HE cell populations is still challenging. Hepatic specification of definitive endoderm (DE) is an essential stage for HE induction in vitro. Recent studies have suggested that circular RNAs (circRNAs) determine the fate of stem cells by acting as competing endogenous RNAs (ceRNAs). To date, the relationships between endogenous circRNAs and hepatic specification remain elusive.

**Methods:**

The identities of DE and HE derived from hiPSCs were determined by qPCR, cell immunofluorescence, and ELISA. Differentially expressed circRNAs (DEcircRNAs) were analysed using the *Arraystar Human circRNA Array*. qPCR was performed to validate the candidate DEcircRNAs. Intersecting differentially expressed genes (DEGs) of the GSE128060 and GSE66282 data sets and the DEcircRNA-predicted mRNAs were imported into *Cytoscape* for ceRNA networks. Gene Ontology (GO) and Kyoto Encyclopedia of Genes and Genomes (KEGG) were involved in the enrichment analysis. Hepatic markers and Wnt/β-catenin were detected in hsa_circ_004658-overexpressing cells by western blotting. Dual-luciferase reporter assays were used to evaluate the direct binding among hsa_circ_004658, miRNA-1200 and CDX2. DE cells were transfected with miR-1200 mimics, adenovirus containing CDX2, and Wnt/β-catenin was detected by western blotting.

**Results:**

hiPSC-derived DE and HE were obtained at 4 and 9 days after differentiation, as determined by hepatic markers. During hepatic specification, 626 upregulated and 208 downregulated DEcircRNAs were identified. Nine candidate DEcircRNAs were validated by qPCR. In the ceRNA networks, 111 circRNA–miRNA–mRNA pairs were involved, including 90 pairs associated with hsa_circ_004658. In addition, 53 DEGs were identified among the intersecting mRNAs of the GSE128060 and GSE66282 data sets and the hsa_circ_004658-targeted mRNAs. KEGG and GO analyses showed that the DEGs associated with hsa_circ_004658 were mainly enriched in the WNT signalling pathway. Furthermore, hsa_circ_004658 was preliminarily verified to promote hepatic specification, as determined by hepatic markers (AFP, ALB, HNF4A, and CK19) (*p* < 0.05*)*. This promotive effect may be related to the inhibition of the Wnt/β-catenin signalling pathway (detected by β-catenin, p-β-catenin, and TCF4) when hsa_circ_004658 was overexpressed (*p* < 0.05). Dual-luciferase reporter assays showed that there were binding sites for miR-1200 in the hsa_circ_004658 sequence, and confirmed the candidate DEG (CDX2) as a miR-1200 target. The level of miR-1200 decreased and the level of CDX2 protein expression increased when hsa_circ_004658 was overexpressed (*p* < 0.05). In addition, the results showed that CDX2 may suppress the Wnt/β-catenin signalling during hepatic specification (*p* < 0.05).

**Conclusions:**

This study analysed the profiles of circRNAs during hepatic specification. We identified the hsa_circ_004658/miR-1200/CDX2 axis and preliminarily verified its effect on the Wnt/β-catenin signalling pathway during hepatic specification. These results provide novel insight into the molecular mechanisms involved in hepatic specification and could improve liver development in the future.

**Graphical Abstract:**

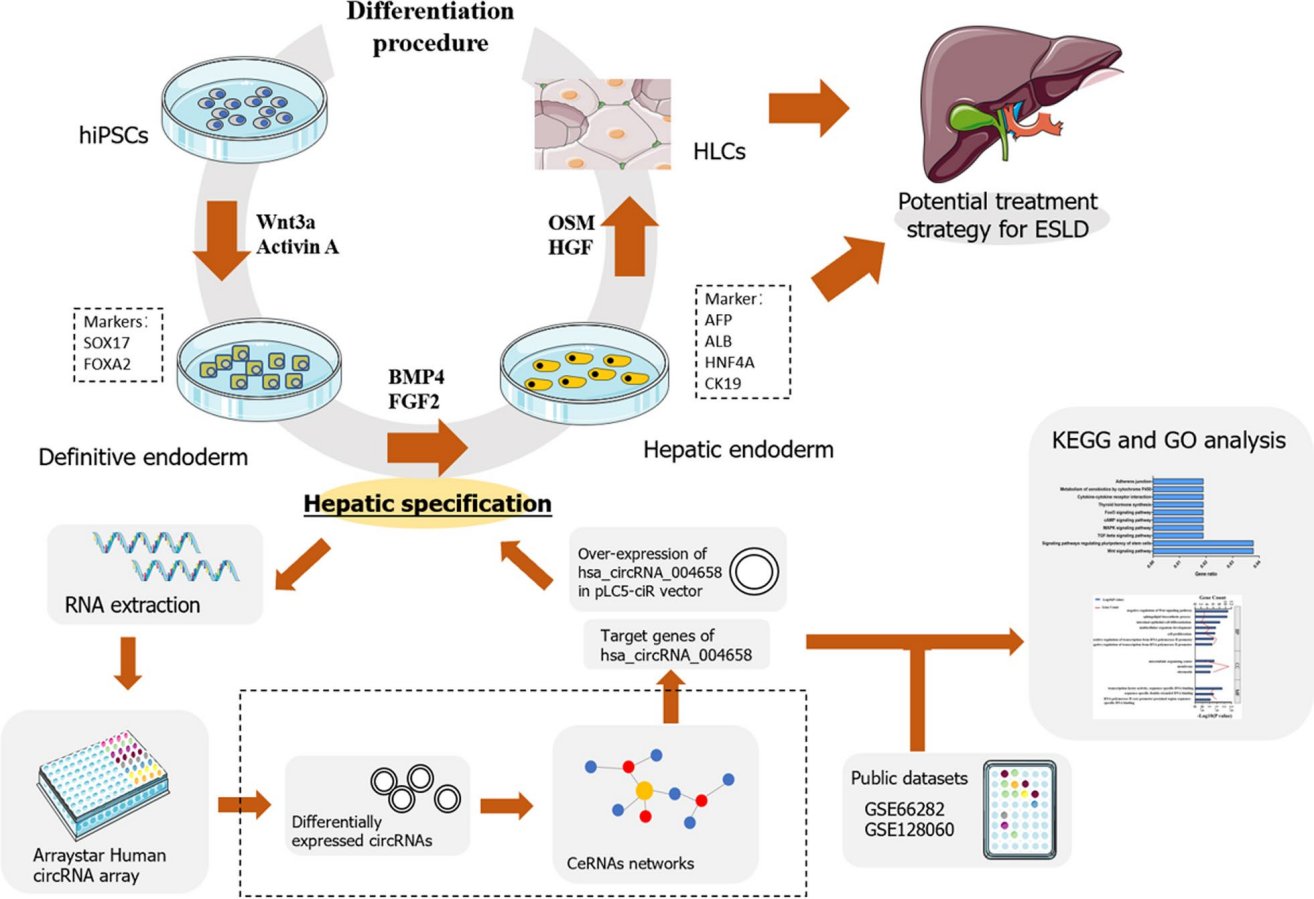

**Supplementary Information:**

The online version contains supplementary material available at 10.1186/s13287-022-03160-z.

## Introduction

Orthotopic liver transplantation is currently one of the most effective strategies to treat end-stage liver disease (ESLD), but it is still limited by donor shortages. Hepatic endoderm (HE), also known as “hepatic progenitor cells”, has the potential to proliferate and differentiate into hepatocytes, cholangiocytes, and hepatobiliary organs both in vitro and in vivo [1–3]. This feature is of particular interest for the development of new treatment strategies for ESLD. In recent years, hepatic progenitor cells have been considered an alternative option for the therapy of liver dysfunctions [4, 5]. HE can be generated by differentiated stem cells from diverse origins, including the directional differentiation of human-induced pluripotent stem cells (hiPSCs), human embryonic stem cells (hESCs), human bone marrow mesenchymal stem cells, and the dedifferentiation of adult hepatocytes/cholangiocytes [6]. Therefore, hiPSCs are a widely used source for obtaining HE with distinctive advantages: (1) resolving the long-standing ethical dispute; (2) reducing the occurrence of rejection reactions during stem cell transplantation; and (3) as pluripotent stem cells obtained from the reprogramming of adult cells, hiPSCs exhibit high similarities with hESCs in terms of cell type. At present, an increasing number of studies have verified the efficacy of HE in repairing liver damage in vivo [2]. Considering that the reprogramming process does not increase the chance of carcinogenesis and genetic mutations, hiPSC-derived HE is expected to improve ESLD more extensively in the future [7].

Hepatic differentiation progresses via an intermediate step, which includes the formation of the definitive endoderm (DE) from iPSCs. This is followed by the generation of HE, and then, their maturation into hepatocyte-like cells (HLCs). This stepwise process follows the natural differentiation process [8]. Hepatic specification, also known as “the process from DE into HE”, is an important process in the formation of HLCs. Research has shown that suppression of the Wnt/β-catenin signalling pathway can promote hepatic specification [9]. Another study demonstrated that overexpressing HNF4A/HHEX or adding specific laminins (culture matrices) may elicit the efficient induction of DE into HE [10–13]. These studies consistently suggested that hepatic specification can be optimized via the regulation of related signalling pathways in vitro to improve the subsequent generation of HLCs.

The majority of circRNAs originate from exonic regions. These circRNAs are broadly expressed during cell development and in a variety of diseases. Basically, circRNAs are in a closed-loop structure and are not easily degraded by RNA exonuclease. Hence, the expression of circRNAs is more stable than that of linear RNAs. Currently, circRNAs have become a research hotspot both in clinical research and molecular biology fields for their potential role in inhibiting the regulatory effects of miRNAs on target mRNAs. Gradually, the increase in our understanding of circRNAs has provided new hope for treating acute and chronic diseases. Sun et al. revealed that circMYBL2 specifically regulates FLT3 kinase levels through translational regulation, providing a potential therapeutic target for acute myeloid leukaemia [14]. In recent years, circRNAs have been suggested to regulate stem cell differentiation and self-maintenance to elicit the differentiation of stem cells into myoblasts, osteoblasts, and cardiomyocytes [15–17]. circMAP3K5 targets the miR-22-3p/TET2 axis and regulates ET2-mediated vascular smooth muscle cell differentiation, which is beneficial for treating intimal hyperplasia [18]. Additionally, circSLC8A1, circCACNA1D, and circSPHKAP have been identified as novel biomarkers for cardiogenesis by applying high-throughput sequencing [19]. In our research, the most interesting finding was that there were large numbers of differentially expressed circRNAs (DEcircRNAs) present in the developmental stages from DE to HE, and these DEcircRNAs may affect the cellular transcriptional profile, cellular function, and even the determination of stem cell fate. Hence, it is important to dissect the regulatory networks and interactions of DEcircRNAs during hepatic specification.

Currently, the mechanism of action by which circRNAs impact hepatic specification remains elusive. Therefore, it is meaningful to analyse the profiles of circRNAs during this process and illustrate the role of specific circRNAs in cell differentiation. At present, there is no research describing the role of circRNAs in the liver cell fate of hiPSCs; thus, this is one of the innovative highlights in our work. In our study, the expression profiles of circRNAs in DE and HE were analysed by Arraystar Human Array. Competing endogenous RNA (ceRNA) networks were constructed to investigate the relationships among DEcircRNAs, miRNAs, and mRNAs. In addition, the intersection of differentially expressed genes (DEGs) from public data sets (accession number: GSE66282 and GSE128060 obtained from Gene Expression Omnibus) (mRNA array) and DEcircRNA-predicted mRNAs were analysed through Gene Ontology (GO) and Kyoto Encyclopedia of Genes and Genome (KEGG). In vitro experiments were performed for preliminary verification. Our study analyses the profiles of circRNAs during hepatic specification. In addition, we preliminarily validated that hsa_circ_004658 overexpression may suppress the Wnt/β-catenin pathway and promote the process of hepatic specification. In addition, we also showed that hsa_circ_004658 could regulate the Wnt/β-catenin signalling pathway by directly sponging miR-1200/CDX2. These results may help to improve hepatic differentiation efficiency and provide a novel research direction for stage-specific circRNAs during hepatic specification.

## Materials and methods

### Cell culture

hiPSCs (ATCC ACS-1011) were cultured on vitronectin XF (*STEMCELL Technologies*)-coated plates obtained from mTeSR1 medium (*STEMCELL Technologies*). Before the initiation of liver differentiation, hiPSCs were enzymatically dissociated into clamps by using 1 × TrypLE Express Enzyme (*Gibco*). The clamps were plated onto vitronectin XF-coated 6-well plates in mTesR1 medium to reach approximately 70% confluence in the following days.

### Hepatic differentiation

The differentiation protocol used to induce the differentiation of hiPSCs, DE cells, HE cells, and HLCs was based on previous research with some modifications [8]. Briefly, during the induction of hiPSCs to DE cells, hiPSCs were cultured in RPMI 1640 medium (*Sigma*), which contained 100 ng/ml Activin A (*STEMCELL Technologies*), 25 ng/ml Wnt3a (*R&D Systems*), and 1 × B27 supplement without vitamin A (*STEMCELL Technologies*), for 4 days. For the induction of DE cells into HE cells, iPSC-derived DE cells were cultured in RPMI 1640 medium containing 20 ng/ml bone morphogenetic protein (BMP4) (*STEMCELL Technologies*), 10 ng/ml fibroblast growth factor 2 (FGF2) (*R&D Systems*), and 1 × B27 supplement without vitamin A for 5 days. HE cells were cultured for 5 days in hepatocyte culture medium (*HCM*, *Lonza*) without epidermal growth factor (EGF), which contained 20 ng/ml hepatocyte growth factor (HGF) (*STEMCELL Technologies*). Finally, the cells were cultured for 11 days in HCM without EGF but with 20 ng/ml oncostatin M (OsM) (*STEMCELL Technologies*). Periodic acid-Schiff (PAS) (*Beyotime*) staining was conducted for hepatocyte function detection following the instructions [20] after 25 days of differentiation.

### CircRNA microarray

After extraction and purification, total DE and HE RNA (three samples in each group) were analysed using a *Human Arraystar circRNA Array* (*Arraystar*). After digestion with RNase R, RNA was amplified and transcribed into fluorescent cRNA utilizing a random priming method (Arraystar Super RNA Labelling Kit). These arrays were analysed by the *Agilent Microarray Scanner* G2505C (Agilent Technologies). Then, Agilent Feature Extraction software (version 11.0.1.1) was used to access the obtained array images.

### qRT–PCR

DEcircRNAs were defined as differentially expressed when *p* < 0.05 with fold change > 1.5 or < − 1.5. In total, 6 upregulated and 3 downregulated candidate circRNAs were validated by qRT–PCR between the DE and HE stages. The sequences of circRNA-specific primers are listed in Table 1. RNA was extracted from the cell samples (three samples in each group). RNA was reverse transcribed into cDNA using a cDNA Reverse Transcription Kit (*Takara*). Divergent primers were used for the analysis of circRNAs. qRT–PCR was performed in a 20 μL reaction system, and GAPDH was used as an internal control on the *Roche LightCycler® 480* sequence detection system. All experiments were performed in triplicate, and relative gene expression is presented as the fold change using the 2^−ΔΔCt^ method. A probability value below 0.05 (*p* < 0.05) was considered statistically significant. In addition, differentially expressed mRNAs between the DE and HE stages (SOX17, FOXA2, AFP, ALB, CK19, and HNF4A) were validated by qRT–PCR, and the primers were designed according to a reference [21].

**Table 1:** PCR Primer lists

circRNAs	Primer sequence	Annealing temperature (℃)	Length (bp)
GAPDH(HUMAN)	F:5′GGGAAACTGTGGCGTGAT3′ R:5′GAGTGGGTGTCGCTGTTGA3′	60	299
hsa_circRNA_004658	F:5′ GCCATCAGCAAATAATGAACCCA3′ R:5′ CGTCCTTGTGAGTCGAAGAACCT3′	60	169
hsa_circRNA_075671	F:5' ACCAGCATCAGAACAGTCAGAGAA3' R:5′ ACCACGGAATCCCATCACTGT3′	60	133
hsa_circRNA_006919	F:5′ CAAACTCGTGGCAATATGTG3′ R:5′ CTGCCCAGTCCAGTCCTAT3′	60	285
hsa_circRNA_104101	F:5′ GGAGCAACAGTAGAAAGTTCTCTA3′ R:5′ GGCACCTTCATCAGTAGTCATT3′	60	56
hsa_circRNA_104981	F:5' GCGCTATGACAAATTGAAGATA3' R:5′ TTCTGACTCCAGTGTTTCCATC3′	60	96
hsa_circRNA_102700	F:5' CCAGAATGATGAAATTGTTAGGT3' R:5′ TAATCGCCGCATGTTGTAC3′	60	63
hsa_circRNA_005232	F:5′ ATCGTTCCATATAAAACCATCG3′ R:5′ CTTCCAACTGTCACAACACAAT3′	60	115
hsa_circRNA_002415	F:5′ GGAGCATGAGGATGAGAAGATA3′ R:5′ CAGTTCTGACTCCAGTGTTTCC3′	60	99
hsa_circRNA_104730	F:5′ AAGCCAAAGCAGCAGGAGTT3′ R:5′ GGTGTGGGTTATAAGCCTTTCC3′	60	68

### CeRNA networks

The interaction between circRNAs and miRNAs was predicted with miRNA targeting prediction software (Arraystar’s home-made) based on TargetScanHuman 7.1 and miRanda. In addition, miRDB, CircBase, and CircBank were involved in circRNA-miRNA analysis. ceRNA networks were constructed based on the correlation between circRNAs-miRNAs and miRNAs-mRNAs by using *Cytoscape 3.9.0* software.

### GO and KEGG analysis

The public data sets GSE128060 (acquired on an Illumina HiSeq 2500) and GSE66282 (acquired on an Illumina HumanHT-12 V4.0 expression beadchip) were obtained from the Gene Expression Omnibus (https://www.ncbi.nlm.nih.gov/geo/). GSE128060 contains 24 samples of endoderm cells (from GSM3661506 to GSM3661529) and 59 samples of hepatic endoderm cells (from GSM3661530 to GSM3661588). GSE66282 contains 2 samples of DE cells (GSM1618660, GSM1618661) and 2 samples of HE cells (GSM1618662, GSM1618663). Series matrix microarray data were processed using the Bioconductor R package (10.18129/B9.bioc.ArrayExpress). Differential expression analysis was performed by the Bioconductor package edgeR (10.18129/B9.bioc.edgeR), and significantly changed genes were chosen according to a significance level of* p* < 0.05 (changed genes are listed in Additional file 1: Appendix 1). The intersecting mRNAs from the GSE128060 data set, GSE66282 data set and hsa_circ_004658-targeted mRNAs were screened out for follow-up research. Gene ontology (GO) was performed using DAVID 2021 (Database for Annotation, Visualization, and Integrated Discovery) software (http://david.niaid.nih.gov/david). Kyoto Encyclopedia of Genes and Genome (KEGG) was performed on the KEGG pathway database (https://www.kegg.jp/kegg/pathway.html).

### Plasmid construction and transfection

The hsa_circ_004658 overexpression plasmid (hsa_circ_004658-OE) was generated by inserting the full length of the hsa_circ_004658 sequence into the pLC5-ciR vector (*Geneseed Biotech*). The hsa_circ_004658-OE vector was designed and then transfected into DE cells with Lipofectamine reagent (*Invitrogen*) according to the manufacturer’s instructions. After 50–60 h of transfection, proteins were extracted from DE-derived cells for further detection. Adenovirus vector (GV345-CDX2, with CDX2 overexpression) construction and packaging were performed by *GeneChem Co., Ltd*. DE cells were transfected with GV345-CDX2 or GV345-vector for approximately 48–72 h, and then, the expression of CDX2 was detected by western blotting.

### Western blotting

Cells were mixed with radioimmunoprecipitation assay (RIPA) buffer (*Solarbio*), phenyl methyl sulfonyl fluoride (PMSF) buffer (*Solarbio*), and phosphatase inhibitors (*Glpbio*) for protein extraction. Protein was electrophoresed using SDS–PAGE (10%, 12%, or 15%) and transferred to nitrocellulose membranes. The membranes were blocked with skim milk (5%) in TBST for 1 h at room temperature and incubated with goat anti-rabbit polyclonal β-catenin, TCF4, AFP, ALB, CK19, β-actin (diluted 1:1000–1:10,000) *(Proteintech*) and p-β-catenin and HNF4A antibodies (diluted 1:1000–1:5000) *(Abcam*) and CDX2 (diluted 1:1000) *(Cell Signal Technology*) at 4 °C overnight. After washing with TBST three times, the nitrocellulose membranes were incubated with a 1:1000 dilution of horseradish peroxidase (HRP) goat anti-rabbit IgG (*Cell Signalling Technology*) for 1 h at room temperature. After washing with TBST three times, the membranes were incubated with a mixture of peroxide solution (*Bio-Rad*) and luminol enhancer solution (*Bio-Rad*) (1:1). Finally, the results were detected by a *Tanon 5500 chemiluminescent imager system* (*Tianneng*) and standardiz to β-actin protein. Western blotting experiments were repeated three times.

### Cell immunofluorescence

Cells cultured on 6-well plates (1000 μL/well)/12-well plates (400 μL/well) were divided into different groups. After washing with phosphate-buffered saline + 0.05% Tween (PBST) three times (5 min each time), the cells were fixed with precooled methyl alcohol for 20 min at − 20 °C. Triton X-100 (0.5%) was added to the cell slide and incubated for 20 min for transparency. After that, the cells were washed with PBST and blocked with bovine serum albumin (BSA) (*Abcone*) for 30 min. Finally, the cells were incubated overnight with SOX17 (Rabbit, *Proteintech*, 1:100)/FOXA2 (Mouse, *Abcam*, 1:200), HNF4A (Rabbit, *Abcam*, 1:200)/AFP (Mouse, *ABclonal*, 1:100), CK19 (Rabbit, *Proteintech*, 1:100)/ALB (Mouse, *Proteintech*, 1:100), or β-catenin antibody (Rabbit, *Proteintech*, 1:100) at 4 °C. After washing 3 times, the cells were incubated with goat anti-mouse/rabbit IgG-FITC or goat anti-mouse/rabbit IgG-RBITC fluorescent secondary antibodies (*Earthox*, 1:500) for 60 min. Then, the cells were mixed with DAPI reagent for 10 min. Cell slides were observed under an optical microscope (MF52-N, *Mshot*) or with Axio Imager M2 software (*Nikon*) (×100, ×200 magnification).

### Enzyme-linked immunosorbent assay

The cell culture supernatant was collected for ELISA detection. The standards and samples, washing buffer, diluted antibody solution, and diluted HRP solution were prepared before detection. First, 100 µL of standard/samples was added to each well, incubated for 120 min and washed 4 times with wash buffer (1×) using at least 500 µL in each well. Then, 100 µL of diluted antibody solution was added, incubated for 60 min, and washed 4 times with wash buffer. Then, 100 µL of diluted HRP solution was added. After a 40-min incubation, it washed 4 times with wash buffer. TMB substrate and stop solution were subsequently added. The absorbance at 450 nm was immediately detected after adding the stop solution. The ELISA experiments were repeated three times.

#### Dual-luciferase reporter assay

The wild-type (wt) and mutant (mut) hsa_circ_004658 or CDX2-3′UTR fragments containing miR-1200 binding sites were fused into the Renilla luciferase gene (*hRluc*) included in the psiCHECK2 vector (*Promega*) using the Xho I and Not I restriction sites. The recombinant vector and miR-1200 mimics or mimics NC were co-transfected simultaneously into DE target cells using Lipofectamine 2000 (*Invitrogen*). Luciferase activity was determined 48 h after transfection using a dual-luciferase reporter assay kit (*Promega*).

#### Statistical analysis

Quantitative data are presented as the mean ± SD from three independent records. The data were analysed by *Student’s t test*, *one-way ANOVA* and *two-way ANOVA* with *SPSS* 20.0 software and *GraphPad Prism 7.0*. *p* < *0.05* was defined as statistically significant. *p < 0.05, **p < 0.01, ***p < 0.001, ****p < 0.0001; ns, not significant.

## Results

### Identification of DE and HE

**Fig. 1:**
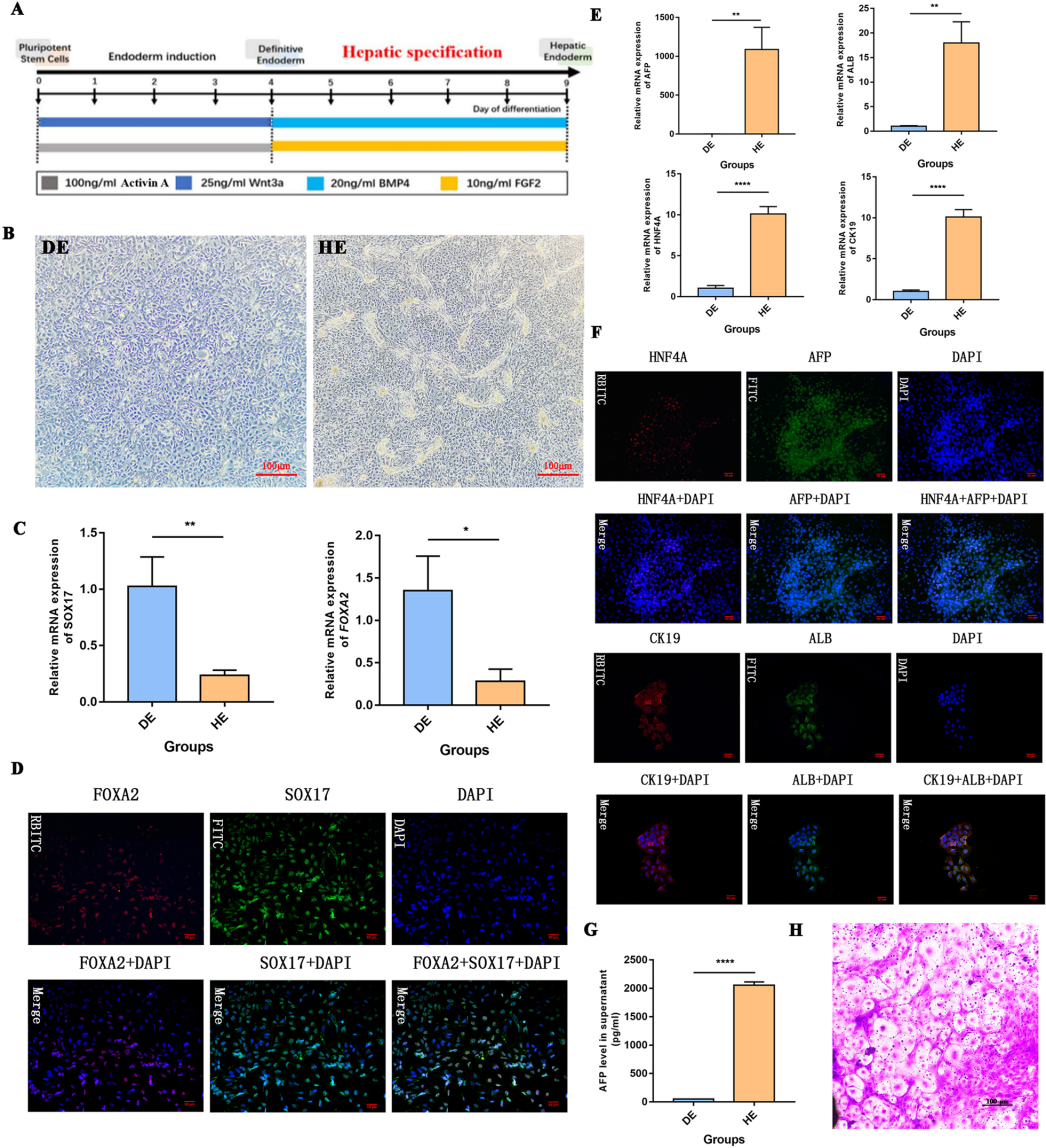
Identification of DE and HE displaying different morphological features and biomarkers. **A** The differentiation protocol for obtaining DE and HE cells from hiPSCs. **B** Morphological observations of DE and HE cells (100×). **C** The expressions of SOX17 and FOXA2 were detected in DE and HE cells by qRT–PCR. **D** Fluorescence signals of SOX17 and FOXA2 were captured in DE cells (200×). **E** The expressions of hepatic markers (AFP, ALB, HNF4A, and CK19) were detected in DE and HE cells by qRT–PCR. **F** Fluorescence signals of AFP, ALB, HNF4A, and CK19 were captured in HE cells (200×). **G** The level of AFP in culture supernatant of DE and HE cells detected by ELISA. **H** PAS staining in hiPSC-derived hepatocyte-like cells after 25 days of differentiation (200 ×). *p < 0.05, **p < 0.01, and ****p < 0.0001

The differentiation protocol (Fig. 1A) was implemented to obtain human DE and HE. Specifically, human iPSCs were cultured in 100 ng/ml Activin A and 25 ng/ml Wnt3a during the endoderm induction stage (0–4 days). After 4 days, when hiPSCs reached the DE stage, 20 ng/ml BMP4 and 10 ng/ml FGF2 were added to the cell culture for hepatic specification (from 4 to 9 days). DE and HE were collected at 4 days and 9 days after differentiation, respectively. After that, an optical microscope was applied for morphological observation. The results showed that cell morphological changes were accompanied by cell differentiation; DE and HE exhibited the typical profile (100×) of cells at each specific stage (Fig. 1B). To further determine DE and HE, qPCR and cell immunofluorescence (RBITC and FITC) were used to detect the markers (SOX17 and FOXA2) of DE and the hepatic markers (AFP, ALB, HNF4A, and CK19) of HE. The results showed high fluorescence signals for SOX17 and FOXA2 in DE cells, which is consistent with the high mRNA levels of SOX17 and FOXA2 (*p* < 0.05) (Fig. 1C, D). The presence of these markers (SOX17 and FOXA2) indicates that the cells were DE cells. In addition, the high fluorescence signals of AFP, ALB, HNF4A, and CK19 was captured in HE cells, and this was accompanied by an increase in the mRNA level of these hepatic markers (*p* < 0.05) (Fig. 1E, F). The presence of these markers (AFP, ALB, HNF4A, and CK19) indicates that the cells were HE cells. Moreover, the ELISA results showed that the AFP expression level in the HE cell supernatant was significantly higher than that in the DE cell supernatant, as shown in Fig. 1G. Glycogen synthesis and hepatocyte function were detected by periodic acid-Schiff (PAS) staining in hiPSC-derived hepatocyte-like cells after 25 days of differentiation (Fig. 1H). These results suggest that we successfully generated DE and HE cells from iPSCs following the recommended differentiation protocol.

### Expression profiles of circRNAs between DE and HE

**Fig. 2:**
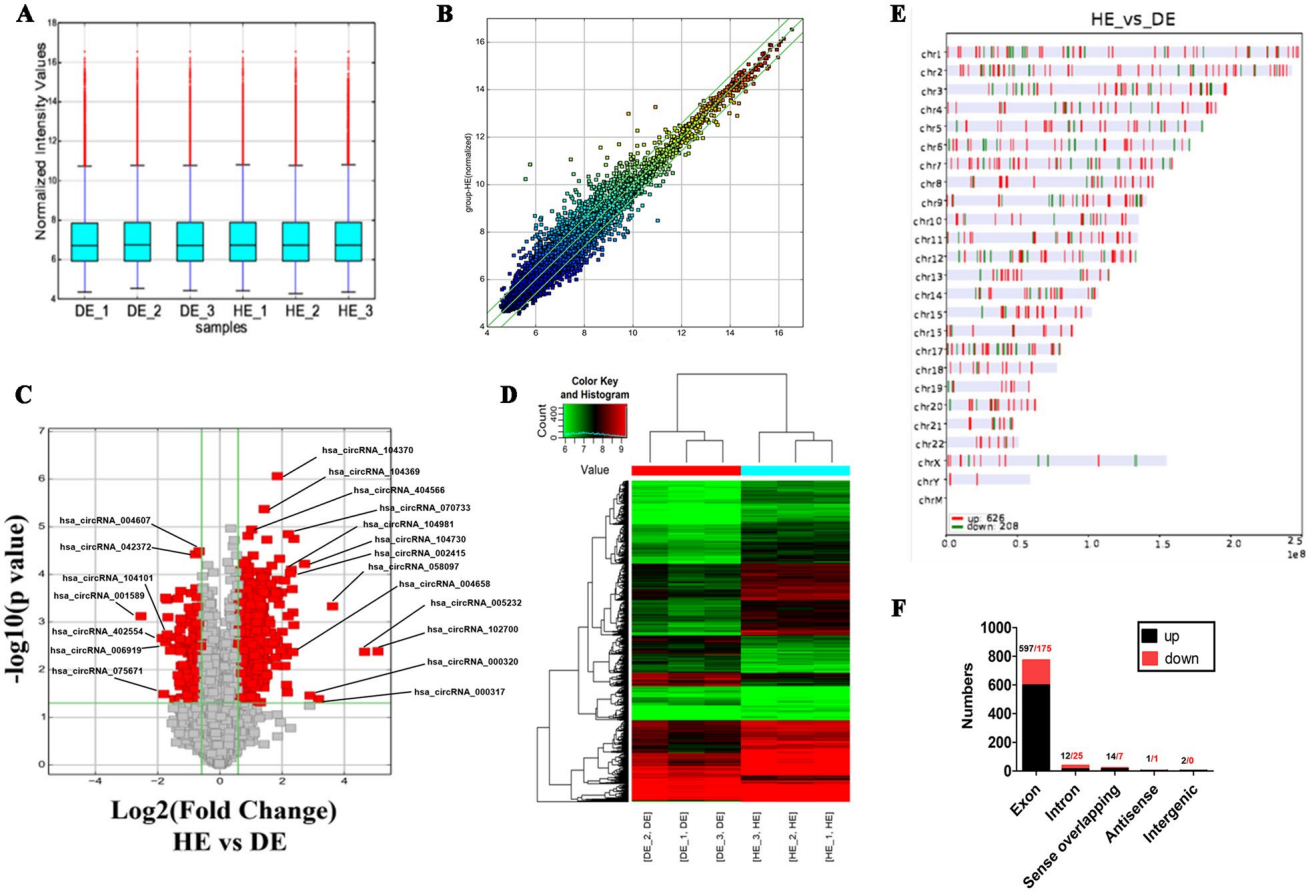
Expression profiles of circRNAs between DE and HE. **A** Normalized intensities of the differentiated cells in the DE and HE groups. **B** Scatter plots for assessing the circRNAs expression variation between DE and HE. (*X* and *Y* represent the averaged normalized signal values of the DE and HE groups, respectively. The middle green line refers to no different circRNAs between the groups. The flanking green lines represent 1.5-fold changes. The points above/below the flanking green lines indicate the circRNAs with 1.5-fold changes between the two groups. **C** Volcano plots for visualizing the identified circRNAs between the DE and HE cells. The red points represent the DEcircRNAs with statistical significance (fold change > 1.5 or < − 1.5, *p* < *0.05*). **D** Hierarchical clustering of DEcircRNAs showing several circRNAs clusters between DE and HE. The red/green matrix represents the upregulated/downregulated circRNAs. **E** The distribution of DEcircRNAs on human chromosomes 1–22, *X*, *Y*, and M. **F** Annotations of the DEcircRNAs (Category: exon, intron, sense overlapping, antisense, and intergenic)

To analyse the expression of DEcircRNAs between DE and HE, we profiled the circRNA population in these stages. In total, 13,466 human circRNAs were obtained by microarray analysis using the *Human Arraystar circRNA Array* and *Agilent Microarray Scanner*. After log transformation and normalization, median-centred values between the DE (3 samples) and HE groups (3 samples) indicate that these data are normalized and cross-comparable, as shown in Fig. 2A. Scatter plots and volcano plots exhibit the circRNA expression variations in identified circRNAs during hepatic specification (Fig. 2B, C), displaying statistical significance (*P* value < 0.05) versus magnitude of change (fold change > 1.5 or < − 1.5). Out of the 13,466 circRNAs on the microarray, 834 DEcircRNAs (fold change > 1.5 or < − 1.5, *p* < *0.05*) between DE and HE were screened out. These included 626 circRNAs (e.g. hsa_circ_102700, hsa_circ_005232, hsa_circ_104730, hsa_circ_104981, hsa_circ_004658, hsa_circ_002415) that were significantly upregulated, and 208 circRNAs (e.g. hsa_circ_006919, hsa_circ_104101, hsa_circ_075671) that were significantly downregulated in HE group (Fig. 2C) compared to the DE group. Moreover, heatmap results display the intravariations (DE group or HE group) and intervariations (between DE and HE groups), indicating the DEcircRNAs during hepatic specification. As shown in Fig. 2D, there was a clear shift in the circRNA expression pattern that was associated with hepatic specification (more details on DEcircRNAs are shown in Additional file 2: Appendix 2. Table S1). In addition, we found that a number of DEcircRNAs were enriched on each chromosome and scaffold, such as chromosome 1 (counts: 63), chromosome 2 (counts: 72), chromosome 3 (counts: 67), and chromosome 4 (counts: 43) (Fig. 2E). Among the upregulated DEcircRNAs, 597 were exonic, 12 were intronic, 14 were sense overlapping, 1 was antisense, and 2 were intergenic. Among the downregulated DEcircRNAs, 175 were exonic, 25 were intronic, 7 were sense overlapping, 1 was antisense, and 0 were intergenic (Fig. 2F).

### qPCR validation of candidate DEcircRNAs

**Fig. 3:**
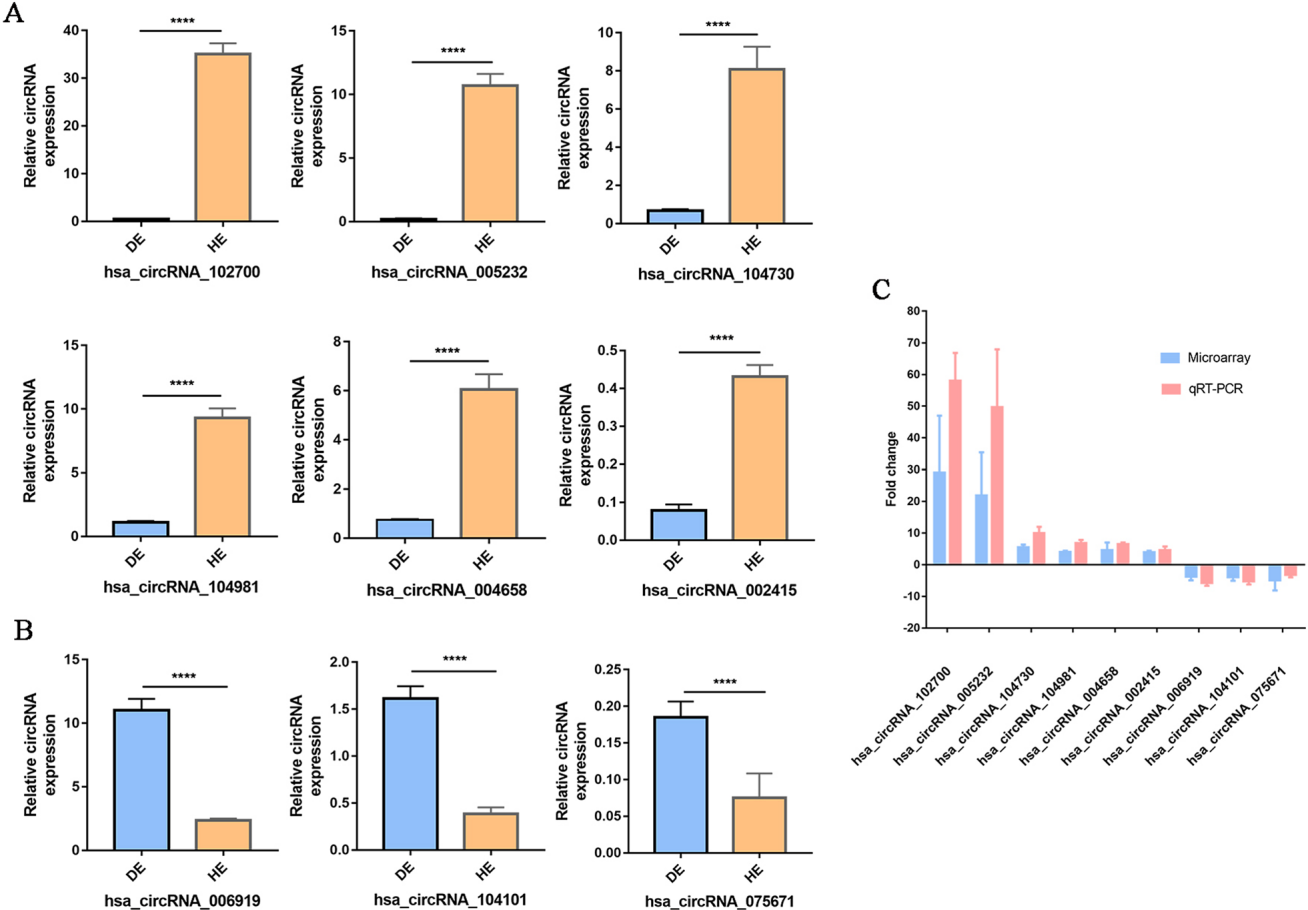
DEcircRNAs between DE and HE were validated by qRT–PCR. **A** The expressions of hsa_circ_102700, hsa_circ_005232, hsa_circ_104730, hsa_circ_104981, hsa_circ_004658 and hsa_circ_002415 were detected by qRT–PCR. **B** The expressions of hsa_circ_006919, hsa_circ_104101 and hsa_circ_075671 were detected by qRT–PCR. **C** Comparisons of microarray results and qRT–PCR results. The results are presented as the means ± SD. ****p < *0.0001*

To confirm the results of the microarray data analysis, qRT–PCR was performed to validate the candidate DEcircRNAs between the DE and HE groups. Among 834 DEcircRNAs (fold change > 1.5 or < − 1.5, *p* < 0.05), 9 candidate DEcircRNAs (6 upregulated DEcircRNAs and 3 downregulated DEcircRNAs) that were abundantly expressed in HE/DE cells were selected for PCR validation. Primers were designed by using *Primer 5.0* and checked for specificity (Table 1). The results showed that hsa_circ_102700, hsa_circ_005232, hsa_circ_104730, hsa_circ_104981, hsa_circ_004658 and hsa_circ_002415 were expressed at higher levels in HE cells than in DE cells, and they increased by 58.08-, 47.34-, 9.86-, 6.78-, 6.53- and 4.49-fold, respectively (*p* < 0.0001) (Fig. 3A). hsa_circ_006919, hsa_circ_104101, and hsa_circ_075671 were expressed at much lower levels in HE, and they were decreased by 3.49-, 3.23-, and 3.00-fold, respectively (*p* < 0.0001) (Fig. 3B). According to our qRT–PCR results, we validated that 6 candidate DEcircRNAs were significantly upregulated in HE cells, and 3 candidate DEcircRNAs were significantly downregulated in HE cells. Note that the expression levels of circRNAs detected by qRT–PCR were consistent with those obtained in the comparative microarray hybridization analysis (Fig. 3C).

### CircRNA-miRNA–mRNA networks

**Fig. 4:**
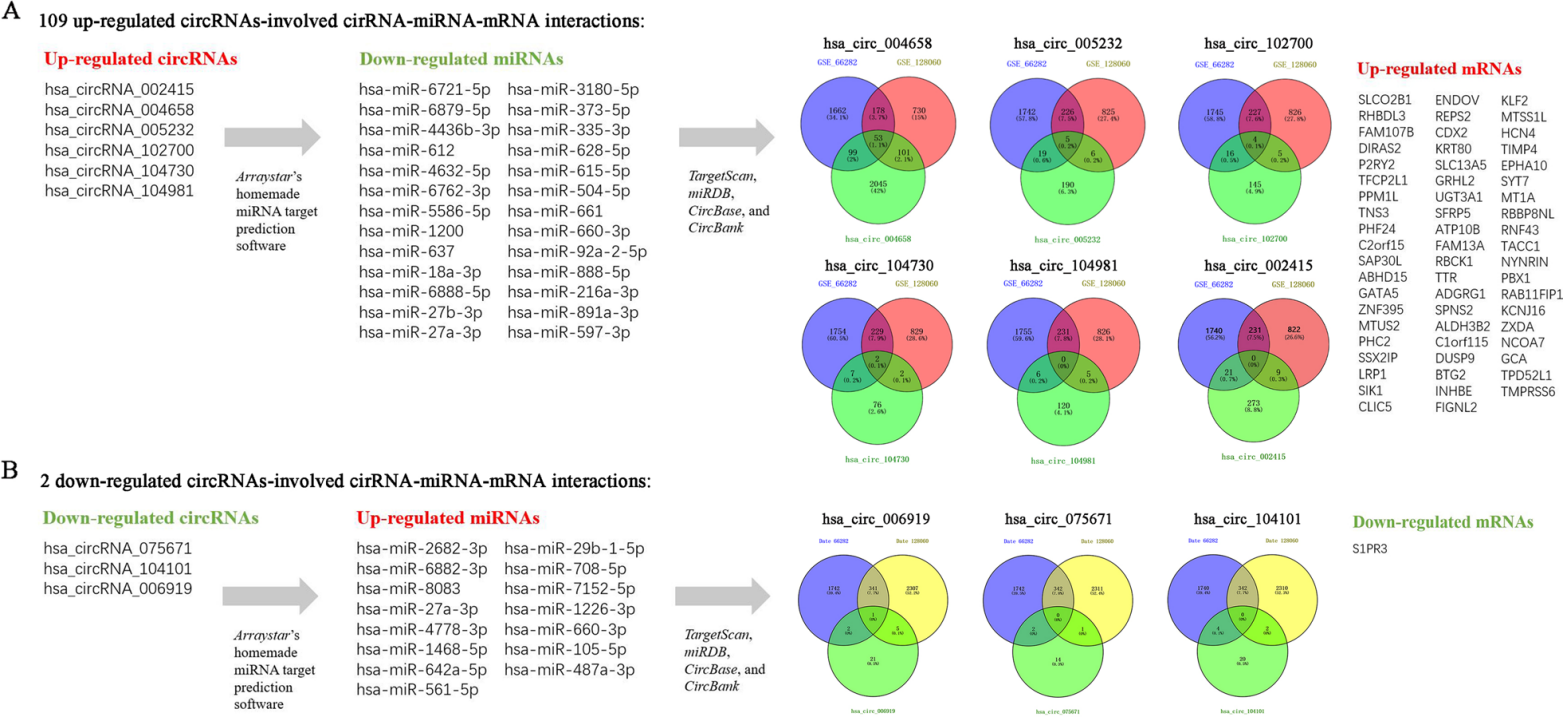
Intersecting DEGs of the GSE128060 and GSE66282 data sets and the DEcircRNA-predicted mRNAs during hepatic specification. **A** Venn diagram of intersecting DEGs targeted by upregulated circRNAs. **B** Venn diagram of intersecting DEGs targeted by downregulated circRNAs

**Fig. 5:**
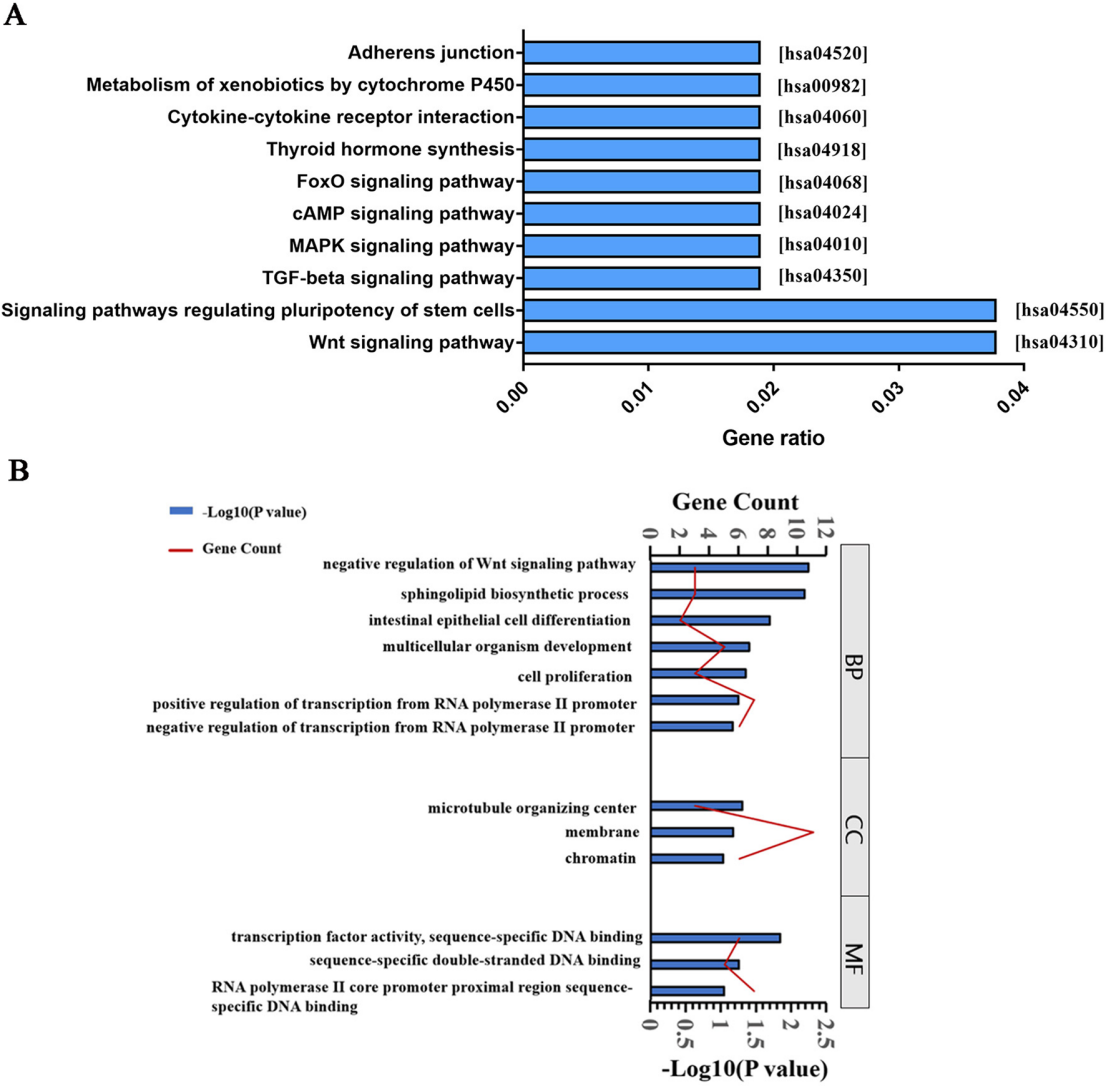
KEGG and GO analysis of the intersecting DEGs interacting with hsa_circ_004658. **A** Top 10 significant KEGG pathways of the target genes. **B** The distribution of the ‘high-rank’ gene ontology during hepatic specification (including biological process, cellular component, and molecular function)

*TargetScan* and *miRanda* were used for predicting the miRNAs that target circRNAs by surveying for 7-mer/8-mer complementarity to the seed region (Additional file 3: Appendix 3.). *TargetScan*, *miRDB*, *CircBase*, and *CircBank* were used to predict the miRNA-targeted mRNAs. Intersecting DEGs of the GSE128060 and GSE66282 data sets and the DEcircRNA-predicted mRNAs were screened for subsequent analysis. As shown in Fig. 4A, 53 intersection mRNAs were obtained in the Venn diagram analysis of hsa_circ_004658 (e.g. LRP1, RNF43, CDX2, SFRP5, etc.). Five intersection mRNAs were obtained in the hsa_circ_005232 group (e.g. DIRAS2, ZXDA, NCOA7, GCA, etc.). Four mRNAs and two mRNAs were found in circ_102700 and circ_104730, respectively. Venn diagram analysis of hsa_circ_006919, hsa_circ_075671, and hsa_circ_104101 is shown in Fig. 4B. Furthermore, the intersecting DEGs of hsa_circ_004658 were also subjected to KEGG pathway analysis and were enriched in the WNT signalling pathway (hsa04310), signalling pathways regulating pluripotency of stem cells (hsa04550), TGF-beta signalling pathway (hsa04350), and MAPK signalling pathway (hsa04010) (Fig. 5A). Interestingly, an interaction between hsa_circ_004658 and hepatic specification was indicated. For this purpose, 53 intersecting mRNAs of hsa_circ_004658 were subjected to DAVID 2021 for GO enrichment analysis in terms of biological process, cellular component, and molecular function. As shown in Fig. 5B, the intersecting mRNAs of hsa_circ_004658 were enriched in “negative regulation of WNT signalling pathway (−Log10Pvalve: 2.22)”, “sphingolipid biosynthetic process (− Log10Pvalve: 2.17)”, “intestinal epithelial cell differentiation (− Log10Pvalve: 1.68)” and “multicellular organism development (− Log10Pvalve: 1.39)”. To a certain extent, these enrichment results are perfectly matched with the differentiation process from DE to HE. Both GO and KEGG showed that the WNT signalling pathway is the most relevant pathway that is associated with hsa_circ_004658 (Fig. 5). Considering that published research has demonstrated a relationship between hepatic specification and the WNT signalling pathway [9, 22], we conducted subsequent experiments to verify the effect of hsa_circ_004658 on the WNT signalling pathway.

**Fig. 6:**
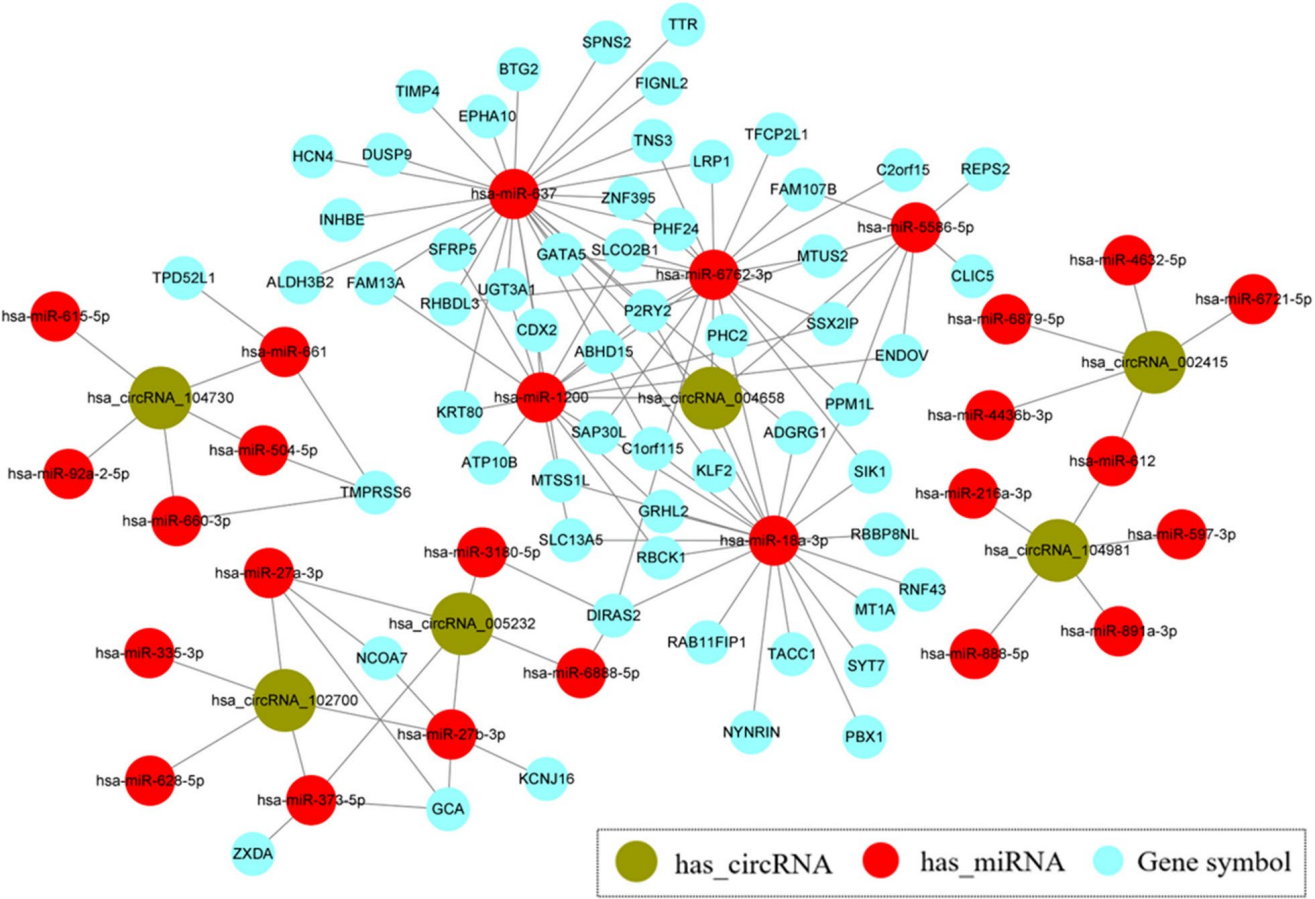
The circRNA–miRNA–mRNA network of hepatic specification. The network of upregulated DEcircRNAs, miRNAs and targeted mRNAs are represented with different shapes (green circle represents hsa_circRNA, red circle represents hsa_miRNA; blue circle represents targeted mRNA)

Five high‐valued miRNA targets were predicted for each candidate DEcircRNA using Arraystar’s home-made miRNA target prediction software. During hepatic specification, 9 validated DEcircRNAs (6 upregulated and 3 downregulated), 45 predicted miRNAs (30 upregulated and 15 downregulated), and 60 targeted mRNAs (59 upregulated and 1 downregulated) were involved in the circRNA–miRNA–mRNA network construction. The network was visualized using *Cytoscape* version 3.9.0. As shown in Fig. 6, we identified 109 circRNA–miRNA–mRNA pairs in the upregulation network during hepatic specification. These included 90 pairs connected to hsa_circ_004658, 9 pairs connected to hsa_circ_005232, and 4 pairs connected to hsa_circ_104730. The results suggested that the candidate DEcircRNAs are active in expression regulation during the differentiation process. In addition, several mRNAs (e.g. SFRP5, LRP1, CDX2) enriched in the WNT signalling pathway were identified in the upregulated network and were predicted to be regulated by multiple circRNAs jointly. These results may provide a novel circRNA-mediated regulatory axis for promoting the induction of DE into HE. The circRNA–miRNA–mRNA network of downregulated circRNAs is shown in Additional file 4: Appendix 4. Figure S1.

### hsa_circ_004658 promotes hepatic specification

**Fig. 7:**
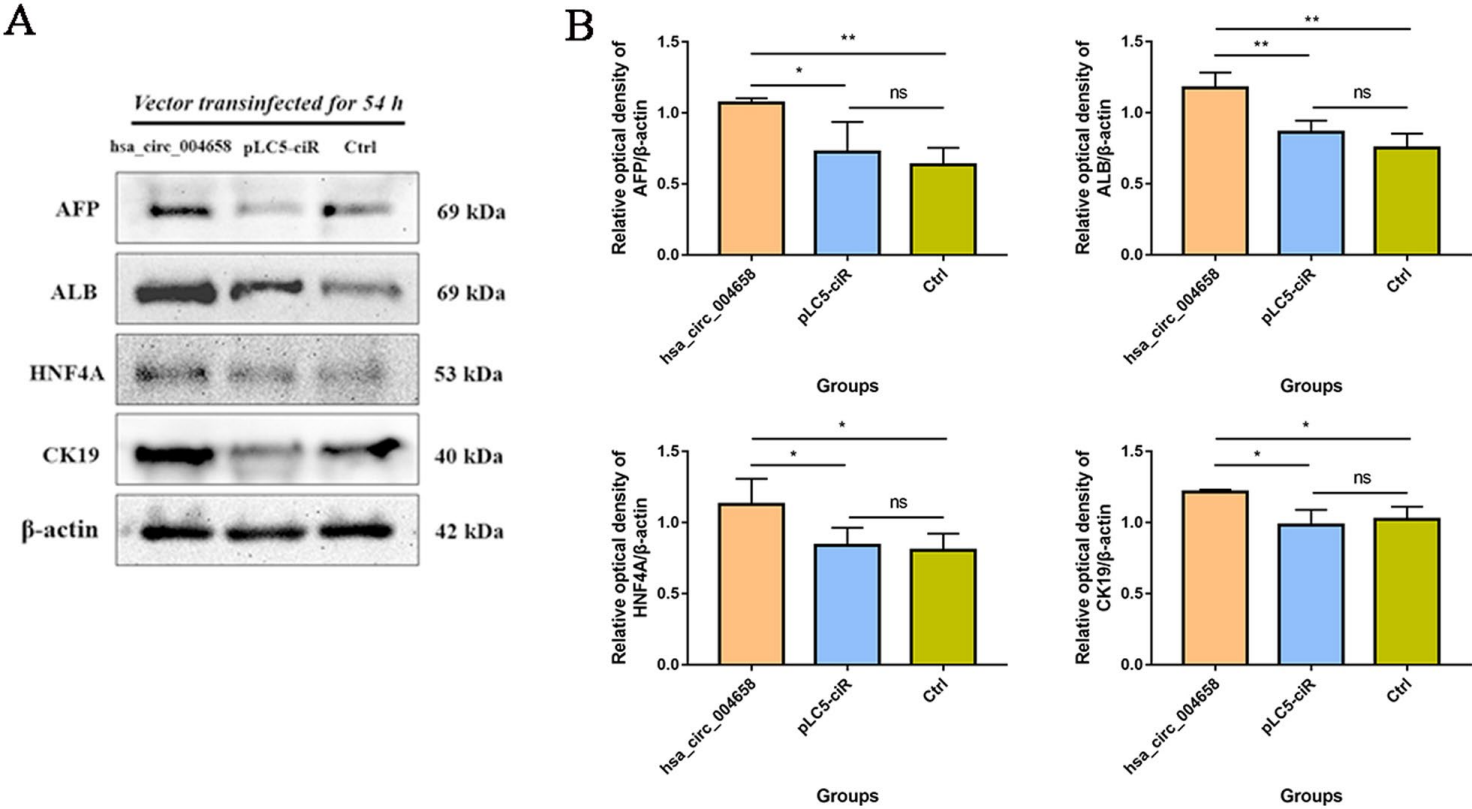
Overexpression of hsa_circ_004658 promotes hepatic specification, as determined by western blotting. **A** Fluorescence intensities of the hepatic markers (AFP, ALB, HNF4A and CK19) in hsa_circ_004658, pLC5-ciR, and control groups. **B** Relative optical densities analysed by *Image J*. Western blotting experiments were repeated three times. *p < 0.05, **p < 0.01; ns, not significant

To determine whether hsa_circ_004658 could affect the differentiation of DE into HE, we overexpressed hsa_circ_004658 iPSC-derived DE cells in vitro. The full length of hsa_circ_004658 (1926 bps) was amplified by PCR and inserted into pLC5-ciR (see Additional file 5: Appendix 5. Figure S2). The constructed vector pLC5-ciR-hsa_circ_004658 (MW: 10.6 kb) was successfully identified by nucleotide sequencing. Efficiency of transfection reached approximately 50% efficiency among the transinfected groups (see Additional file 6: Appendix 6. Figure S3). After overexpression, the relative expression of hsa_circ_004658 dramatically increased in circ_004658 overexpression group, when compared to the pLC5-ciR and control groups (*p* < 0.01) (see Additional file 7: Appendix 7. Figure S4). In addition, the results of western blotting showed that the hepatic markers (AFP, ALB, HNF4A, and CK19) increased significantly in overexpressed cells transfected with the pLC5-ciR-hsa_circ_004658 vector compared to the pLC5-ciR and control groups (*p* < 0.05), while there was no significant difference between the pLC5-ciR group (no-load control vector) and the control group (*p* > 0.05) (Fig. 7A, B). Both hepatic markers (Fig. 7) and AFP level (detected in cell supernatant) (Additional file 8: Appendix 8. Figure S5) consistently suggested that overexpression of hsa_circ_004658 promotes the process of hepatic specification in vitro.

### hsa_circ_004658 directly sponges miR-1200 to regulate the Wnt/β-catenin signalling pathway

**Fig. 8:**
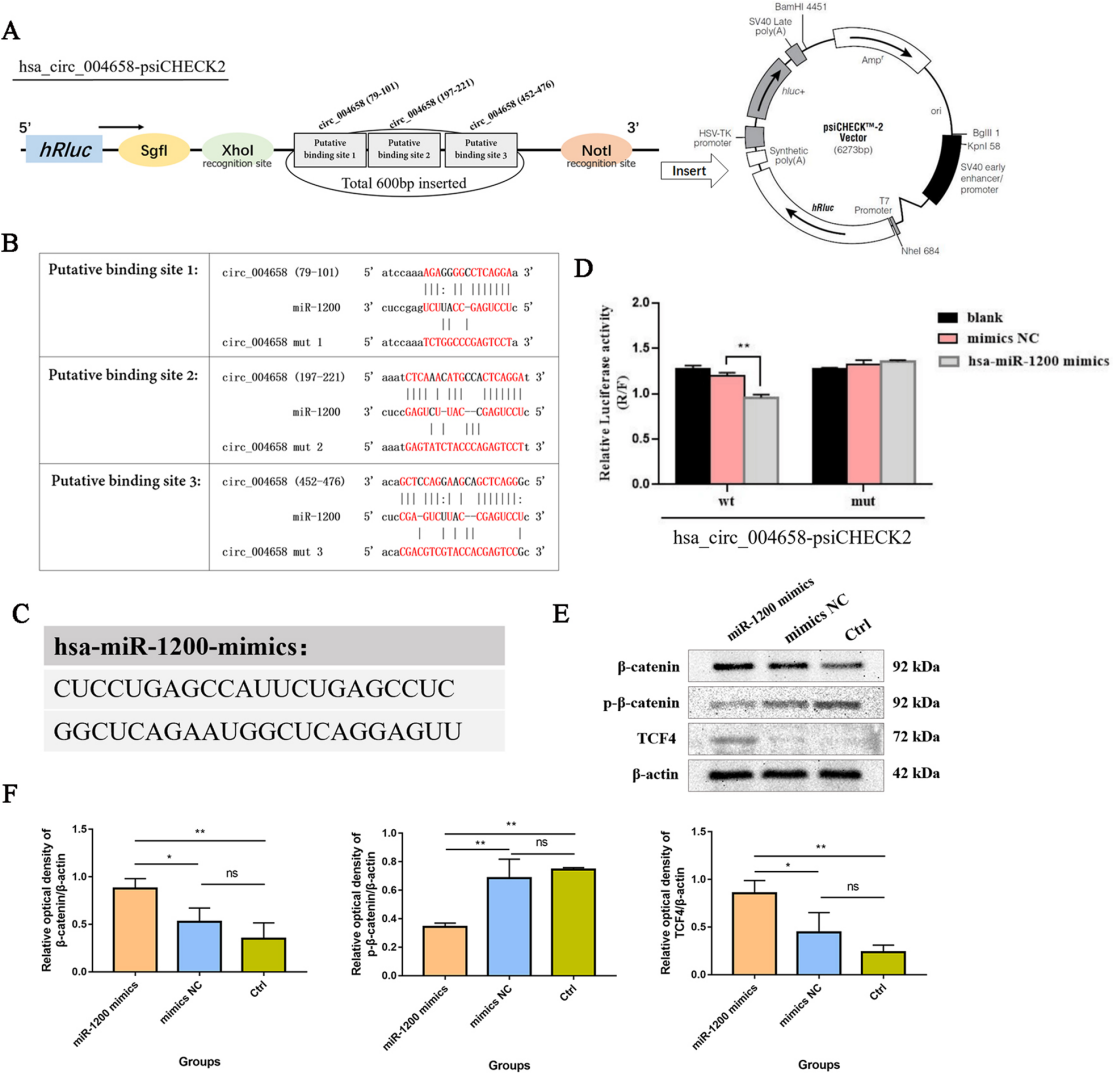
hsa_circ_004658/hsa-miR-1200 axis suppresses the Wnt/β-catenin signalling pathway during hepatic specification. **A** The constructed psiCHECK2 plasmid for dual–luciferase reporter assay. **B** Three putative binding sites between hsa_circ_004658 and miR-1200. **C** The sequences of miR-1200 mimics. **D** Verification of miR-1200 as a target miRNA of hsa_circ_004658 by the dual–luciferase reporter assay. **E** Fluorescence intensities of the markers (β-catenin, p-β-catenin and TCF4) of the Wnt/β-catenin signalling pathway among the miR-1200 mimics, mimics NC and control groups. **F** Relative optical density analysed by *Image J*. Western blotting experiments were repeated three times. *p < 0.05, **p < 0.01; ns, not significant

**Fig. 9:**
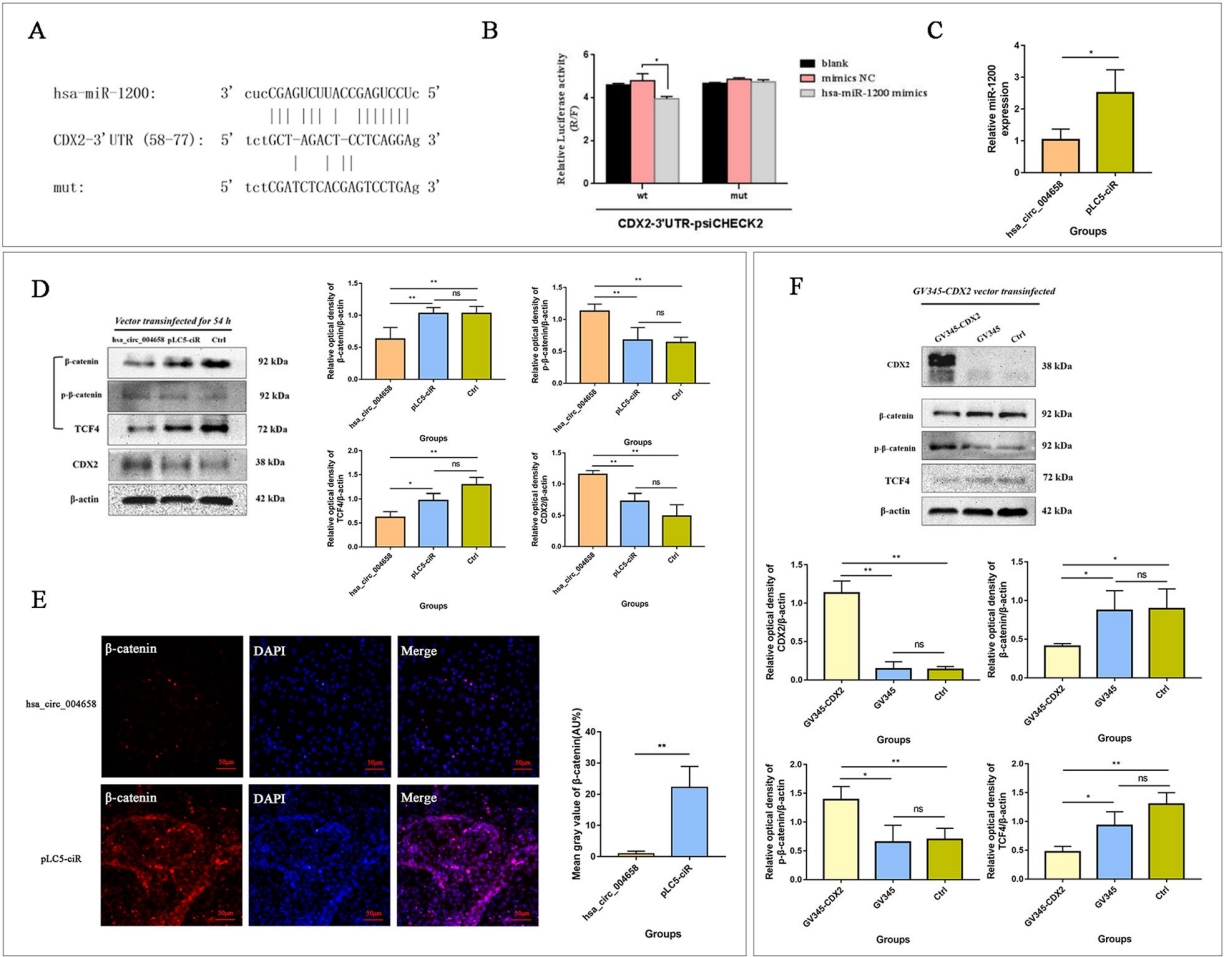
hsa_circ_004658/miR-1200/CDX2 axis suppresses the Wnt/β-catenin signalling pathway, as determined by western blotting and immunofluorescence. **A** The putative binding site between miR-1200 and CDX2. **B** Validation of CDX2 as a miR-1200 target gene by dual-luciferase reporter assay. **C** Relative miR-1200 expression in the hsa_circ_004658 and pLC5-ciR groups. **D** Fluorescence intensities of the markers (β-catenin, p-β-catenin and TCF4) of the Wnt/β-catenin signalling pathway and CDX2 among the hsa_circ_004658, pLC5-ciR and control groups. **E** The fluorescence signal of β-catenin was captured in hsa_circ_004658 and pLC5-ciR groups cells (100×). **F** Fluorescence intensities of the markers (β-catenin, p-β-catenin and TCF4) of the Wnt/β-catenin signalling pathway after CDX2 overexpression. Relative optical density analysed by *Image J*. The experiments were performed three times. *p < 0.05, **p < 0.01; ns, not significant

A dual-luciferase reporter assay was applied to determine the binding effect of circRNAs to targeted miRNAs. The results showed that hsa_circ_004658 could directly bind to miR-1200 at the recognized sites, and 3 putative binding sites of hsa_circ_004658 were inserted into the psiCHECK2 plasmid: binding site 1 (position: 79–101), binding site 2 (position: 197–221), and binding site 3 (position: 452–476) (Fig. 8A, B). Here, for the first time, we preliminarily validated that binding sites for miR-1200 can be found in the hsa_circ_004658 sequence (Fig. 8C, D). On the premise that hsa_circ_004658 overexpression suppresses the Wnt/β-catenin signalling pathway during differentiation, hiPSC-derived DE cells were transfected with miR-1200 mimics in vitro. Furthermore, we detected the Wnt/β-catenin signalling pathway by western blotting. The results showed decreased phosphorylated β-catenin (p-β-catenin) expression and increased total β-catenin/TCF4 expression after treatment with miR-1200 mimics (Fig. 8E, F). Here, miR-1200 mimics exhibited active Wnt/β-catenin signalling, whereas hsa_circ_004658 overexpression displayed the opposite effect (Fig. 9D, E). In summary, hsa_circ_004658 could regulate the Wnt/β-catenin signalling pathway by directly sponging miR-1200, which promotes hepatic specification.

### hsa_circ_004658/miR-1200/CDX2 may suppress the Wnt/β-catenin signalling pathway

In addition, a dual-luciferase reporter assay was conducted to confirm the candidate DEG (CDX2) as a miR-1200 target. The results showed that miR-1200 could directly bind to the CDX2-3’UTR at the recognized site (position: 58–77) (Fig. 9A, B), while the expression of miR-1200 (hsa-miR-1200-RT1: GTCGTATCCAGTGCAGGGTC CGAGGTATTCGCACTGGATACGACGAGGCT; hsa-miR-1200-F1: GCGCTCCT GAGCCATTCTG; Com R: AGTGCAGGGTCCGAGGTATT; U6: U6-F: CTCG CTTCGGCAGCACA; U6-R: AACGCTTCACGAATTTGCGT) decreased when hsa_circ_004658 was overexpressed (*p* < 0.05) (Fig. 9C). Inhibition of the Wnt/β-catenin signalling pathway is considered a strategy for promoting the differentiation of hepatic specification [22–24].Considering the bioinformatics results of GO and KEGG, we hypothesize that hsa_circ_004658 may promote the differentiation of iPSC-derived DE cells into HE cells by affecting WNT signalling pathways. For further exploration, western blotting was conducted to detect the expression of β-catenin, p-β-catenin, and TCF4 in cells in the hsa_circ_004658, pLC5-ciR, and control groups. After transfection with the vector overexpressing hsa_circ_004658 for 54 h, we found increased phosphorylated β-catenin (p-β-catenin) expression and decreased total β-catenin expression in DE-derived cells. These effects were accompanied by the downregulation of TCF4 expression (Fig. 9D, E) (*p* < 0.01). Moreover, a lower fluorescence signal of total/nuclear β-catenin expression was found in hsa_circ_004658 cells compared to pLC5-ciR cells (Fig. 9E). Interestingly, the level of CDX2 protein expression was preliminarily proven to be upregulated when hsa_circ_004658-overexpression in DE-derived cells (Fig. 9D) in this study. For further exploration of CDX2 function, we overexpressed CDX2 in targeted cells by transinfection with the GV345-CDX2 vector and detected the inhibition of the WNT signalling pathway. The results showed that CDX2 overexpression suppressed total β-catenin/TCF4 expression and significantly increased p-β-catenin expression (Fig. 9F) (*p* < 0.05). These results suggest that hsa_circ_004658/has-miR-1200/CDX2 may affect the expression of β-catenin/TCF4 and inhibit the Wnt/β-catenin signalling pathway during hepatic specification. These results are consistent with our GO and KEGG analyses (enriched in “negative regulation of WNT signalling pathway”).

## Discussion

According to a surveillance report issued by the World Health Organization (WHO), over 354 million people worldwide live with chronic hepatitis, and more than one million people die from liver disease and cancer every year. The existing treatment strategies are constrained by donor shortages. At present, an increasing number of mechanistic studies aiming to improve the induction of HE and HLCs to promote the theoretical development of in vitro differentiation of the liver have been reported [25, 26]. Foreseeably, hiPSC-derived HE and HLCs may open an avenue for future treatment of ESLD. Since Yamanaka et al. first defined OSKC exogenous reprogramming factors (OCT4, SOX2, KLF4, and c-MYC), this is no longer a barrier in reprogramming embryonic fibroblasts into iPSCs [27]. iPSCs have gradually become a valuable stem cell source in tissue engineering and regenerative medicine fields with the advantages of wide availability and ease of handling. In 2010, Si-Tayeb et al. initially summarized basic molecular mechanisms (described Wnt/β-catenin, TGF-β, MAPK signalling pathway, etc.) that control the formation of the liver in a systematic review [8]. During the differentiation of hiPSCs into HLCs, hepatic specification is considered a crucial step and limits hepatic development [28, 29]. Promoting the process of hepatic specification is an urgent problem that deserves further exploration. In recent years, circRNAs have been deemed to be endogenous molecular sponges of miRNAs that modulating several important biological processes, including stem cell differentiation, tumorigenesis, and chronic hepatitis [30–32]. Based on this new perspective, we believe that circRNAs have the potential to modulate iPSC/ESC differentiation into liver cells, which may offer a novel therapeutic option for the treatment of ESLD. For this purpose, we investigated the profiles of DEcircRNAs between hiPSC-derived DE and HE for the first time, which may help to identify the mechanisms involved in hepatic differentiation from the perspective of circRNAs.

The differentiation from DE to HE is a well-organized process that is precisely guided and characterized by various factors. Previous studies suggested that multiple regulatory factors (BMP4, FGF2, Activin A, WNT3a, etc.) modulate liver development in vitro [33]. By involving transcriptomic analysis, it is highly efficient and convincing to explore the intersection of the DEGs from three datasets (GSE128060, GSE66282, and DEcircRNA-predicted mRNAs) related to hepatic specification. These DEGs change with differentiation and may potentially function as regulators or indicators of hepatic specification. After excluding the irrelevant and modestly expressed genes, a total of 59 upregulated genes (LRP1, RNF43, CDX2, SFRP5, SLCO2B1, TFCP2L1, TTR, KLF2, etc.) were identified between DE and HE. Under the premise that hepatic specification is negatively correlated with Wnt/β-catenin [22, 24], it is reasonable to speculate that RNF43, SFRP5, and LRP1 may serve as modulators of hepatic specification via the Wnt/β-catenin signalling pathway [34–36]. In addition, KLF2 and TFCP2L1 have been proven to act synergistically to induce ESC self-renewal by mediating Wnt/β-catenin [37]. Undoubtedly, Wnt/β-catenin is considered a signalling pathway that plays a dominant role in embryonic development and stem cell differentiation. Inhibition of Wnt/β-catenin significantly promotes the induction of HLCs from endodermal cells triggered by BMP4/FGF2 [9, 22]. The above research points are consistent with our GO and KEGG analyses, suggesting that the occurrence of hepatic specification is accompanied by Wnt/β-catenin signalling suppression.

Furthermore, the upregulated DEGs (gene symbols) were imported into *String 11.5* and *Cytoscape* to construct protein interaction networks. Under the constructed networks based on *the cytoHubba* (maximal clique centrality, MCC) analysis pattern, CDX2 and GATA5 were predicted to function as hub genes (Additional file 9: Appendix 9. Figure S6). Caudal-type homeobox protein 2 (CDX2) is important in an extensive range of functions, from early differentiation to maintenance of digestive tissues. Morris et al. suggested that human skin fibroblasts (HSF) could not be reprogrammed into hepatocytes when CDX2 was deleted, and an increase in the cellular expression of CDX2 could result in acceptable differentiation outcomes [38]. Recent studies proved that CDX2 and its potentially targeted Wnt/β-catenin pathway might play an important role in regulating the differentiation of DE into HE. Hence, we hypothesized that CDX2 might also affect the process of hepatic specification. Similarly, there also exists a close relationship between GATA5 and Wnt/β-catenin in terms of cell growth and proliferation [39]. These discoveries greatly elicit great interest in exploring the regulatory axis of hub genes. It is worth mentioning that CDX2 has been proven to promote hepatic specification in our rescue experiments, which will be published in the future.

CircRNAs have been found to be ubiquitous and diverse during cell differentiation. Increasing evidence has found that circRNAs can serve as regulators of stem cell differentiation by manipulating stemness [40]. Considering that circRNAs are relatively stable and usually expressed in a cell-type manner [41], the profiles of circRNAs during hepatic specification are worthy of analysis. Based on the constructed ceRNA network, multiple circRNA pairs were predicted to be correlated with the WNT pathway, including 2 pairs (hsa_circRNA_004658–miR-1200–CDX2, hsa_circRNA_004658-miR-637–CDX2) that were identified and predicted to modulate CDX2, 2 pairs (hsa_circRNA_004658-miR-18a-3p–KLF2, hsa_circRNA_004658–hsa-miR-637–KLF2) that were predicted to be connected to KLF2, 2 pairs (hsa_circRNA_004658-miR-6762-3p–LRP1, hsa_circRNA_004658–miR-637–LRP1) that were connected to LRP1, 2 pairs (hsa_circRNA_004658-miR-637–SFRP5, hsa_circRNA_004658-hsa_miR_1200-SFRP5) that were connected to SFRP5, and 1 pair (hsa_circRNA_004658-miR-18a-3p–RNF43) that was connected to RNF43. Notably, these circRNA-miRNA–mRNA regulatory axes were successfully predicted in our study and will be beneficial to further mechanistic explorations of liver development. In this study, for the first time, we identified the hsa_circ_004658/miR-1200/CDX2 axis and preliminarily verified its effect on Wnt/β-catenin signalling pathway during hepatic specification.

From another perspective, several WNT-related circRNA–miRNA–mRNA pairs were identified and associated with hsa_circ_004658, which is consistent with our microarray results. To date, no research on hsa_circ_004658 in stem cell differentiation has been reported. To preliminarily verify the potential regulatory role of hsa_circ_004658, the hsa_circ_004658-overexpressing vector was designed and then transfected into DE cells. Western blotting results showed that hepatic markers increased in hsa_circ_004658-overexpressing DE cells, suggesting that overexpression of hsa_circ_004658 promotes the process of hepatic specification. Moreover, we verified that hsa_circ_004658 overexpression might result in the suppression of the WNT pathway. Here, we report, for the first time, that hsa_circ_004658 may be an important circRNA that promotes the process from DE into HE, while a high level of hsa_circ_004658 expression is negatively correlated with the Wnt/β-catenin pathway. In the case of many-to-one or many-to-many relationships, 2970 targeted genes predicted by hsa_circ_004658 were imported into KEGG and enriched in the “MAPK signalling pathway,” “PI3K-Akt signalling pathway,” and “WNT signalling pathway” (Additional file 10: Appendix 10. Figure S7).

However, several issues need to be addressed here. First, hepatic specification is the second step during the in vitro liver development process. According to gene set enrichment analysis (continuous variable) of public data sets, few development-related genes that exhibited continuous changes were identified during the four steps of differentiation. Hence, we focused our attention on its important intermediate step. Second, in this study, we aimed to analyse the profiles of differentially expressed circular RNAs between iPSC-derived DE and HE for the first time. Moreover, the effect of hsa_circ_004658 on hepatic specification was preliminarily verified in vitro, which may be related to the inhibition of the WNT signalling pathway. In addition, we first validated that hsa_circ_004658 could regulate the Wnt/β-catenin signalling pathway by directly sponging miR-1200. Further exploration of such regulatory networks should also be conducted in the future. Third, recently, the CRISPR/Cas13 system has been applied to knock down circRNAs with high specificity and efficiency. The conclusion of this study will be more convincing when CRISPR/Cas13 is used for rescue experimental design [42].

## Conclusions

In summary, this study well analysed the profiles of differentially expressed circRNAs during hepatic specification from hiPSC-derived DE into HE. Combined with the public data sets and circRNA sequence results, 9 validated circRNAs (6 upregulated and 3 downregulated) and 111 circRNA–miRNA–mRNA predicted regulatory pairs (109 upregulated and 2 downregulated) were identified for further research. In addition, hsa_circ_004658 was verified to promote hepatic specification by inhibiting the β-catenin/TCF4 signalling pathway in vitro. Besides, we identified the hsa_circ_004658/miR-1200/CDX2 axis and preliminarily verified its effect on the Wnt/β-catenin signalling pathway during hepatic specification. These results may provide novel insight into the molecular mechanisms involved in liver differentiation and improve the efficient induction of DE into HE in the future.

## Supplementary Information

**Additional file 1**. **Appendix. 1:** Changed genes lists of GSE66282 and GSE128060.

**Additional file 2**. **Appendix 2. Table S1**: Upregulated and downregulated differentially expressed circRNAs during the hepatic specification.

**Additional file 3**. **Appendix 3**: CircRNAs target-miRNAs prediction by using TargetScan and miRanda.

**Additional file 4.** **Appendix 4. Figure S1**: CircRNA–miRNA–mRNA regulatory network during hepatic specification. The network of downregulated DEcircRNAs, miRNAs and targeted mRNAs are represented with different shapes (green circle represents hsa_circRNA, red circle represents hsa_miRNA; blue circle represents targeted mRNA).

**Additional file 5.** **Appendix 5. Figure S2**: Diagram of the constructed vector pLC5-ciR-hsa_circ_004658.

**Additional file 6**. **Appendix 6. Figure S3**: Transfection efficiency of DE cells after transinfected with pLC5-ciR-hsa_circ_004658 (GFP) plasmid.

**Additional file 7**. **Appendix 7. Figure S4**: Relative expression of hsa_circ_004658 in DE cells after transinfected with pLC5-ciR-hsa_circ_004658.

**Additional file 8**. **Appendix 8. Figure S5**: The level of AFP in cell supernatant at 54 hours after trans-infection.

**Additional file 9**. **Appendix 9. Figure S6**: Protein-protein interaction network construction and hub genes screening among upregulated DEGs.

**Additional file 10.** **Appendix 10. Figure S7**: KEGG enrichment analysis of 2970 target-genes predicted by hsa_circ_004658.

## Data Availability

The datasets used and/or analysed during the current study are available from the corresponding author on reasonable request.

## Abbreviations

hiPSCs: Human-induced pluripotent stem cells
hESCs: Human embryonic stem cells
ESLD: The end-stage liver disease
HE: Hepatic endoderm
DE: Definitive endoderm
HLCs: Hepatocyte-like cells
circRNAs: Circular RNAs
miRNAs: Micro RNAs
mRNAs: Messenger RNAs
ceRNAs: Competing endogenous RNAs
DEcircRNAs: Differential expression of circRNAs
qRT-PCR: Quantitative reverse transcription–polymerase chain reaction
DEGs: Differentially expressed genes
GO: Gene ontology
KEGG: Kyoto encyclopedia of genes and genomes
hsa_circ_004658-OE: Hsa_circ_004658 overexpression plasmid
FOXA2: Forkhead box protein A2
SOX17: Sex-determining region Y-box 17
AFP: Alpha-fetoprotein
ALB: Albumin
HNF4A: Hepatocyte nuclear factor 4 alpha
CK19: Cytokeratin 19
β-catenin: Beta-catenin
TCF4: T cell factor 4
BMP4: Bone morphogenetic protein
FGF2: Fibroblast growth factor 2
HGF: Hepatocyte growth factor
OSM: Oncostatin M
BP: Biological process
CC: Cellular component
MF: Molecular function
