# Supplementary material for: Integrative network analysis of circular RNAs reveals regulatory mechanisms for hepatic specification of human iPSC-derived endoderm
Source: Stem Cell Res Ther. 2022 Sep 8;13:468. doi: 10.1186/s13287-022-03160-z (PMC9461288; doi:10.1186/s13287-022-03160-z)

hsa-miR-612_vs_hsa_circRNA_002415

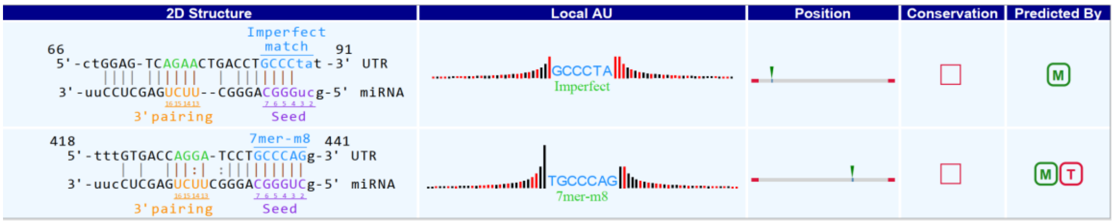

hsa-miR-4436b-3p_vs_hsa_circRNA_002415

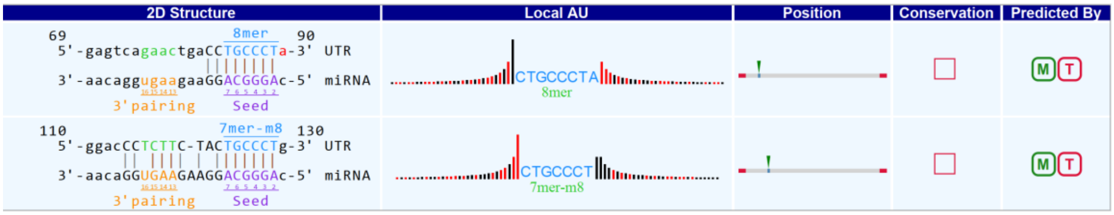

hsa-miR-4632-5p_vs_hsa_circRNA_002415

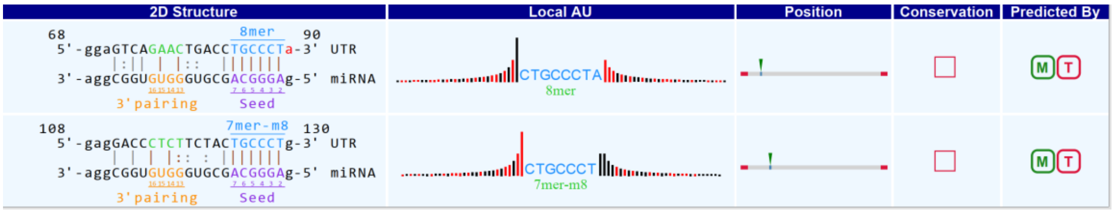

hsa-miR-6721-5p_vs_hsa_circRNA_002415

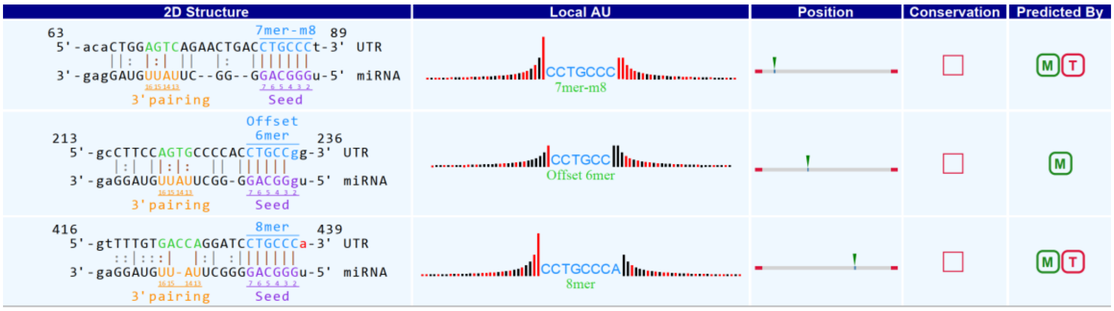

hsa-miR-6879-5p_vs_hsa_circRNA_002415

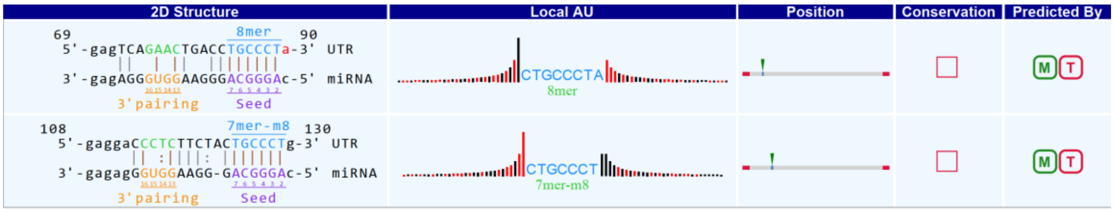

hsa-miR-18a-3p_vs_hsa_circRNA_004658

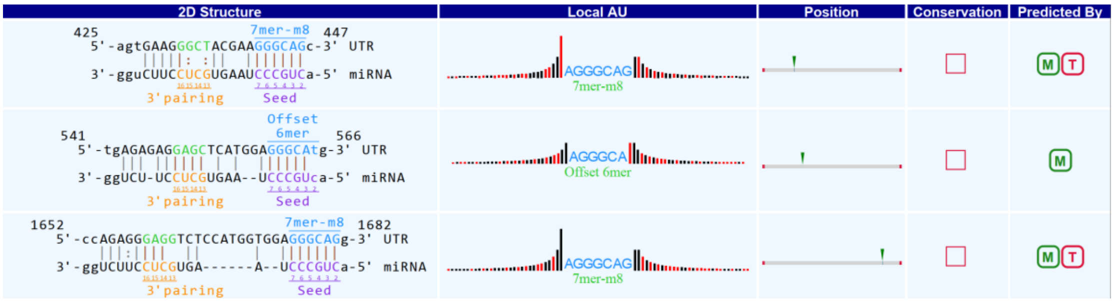

hsa-miR-637_vs_hsa_circRNA_004658

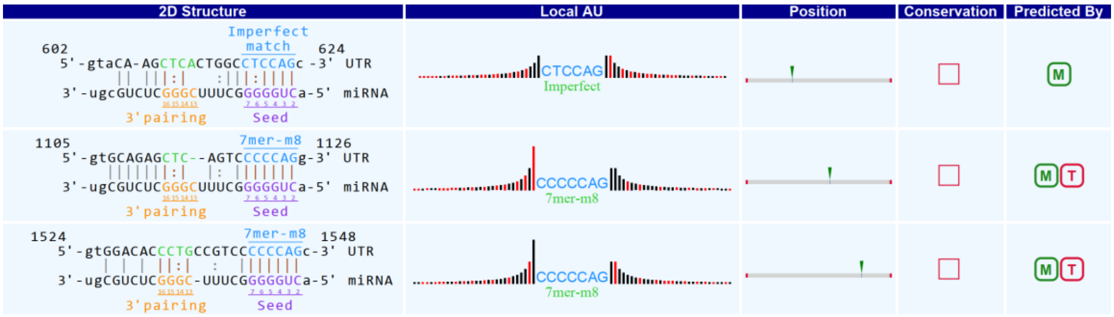

hsa-miR-1200_vs_hsa_circRNA_004658

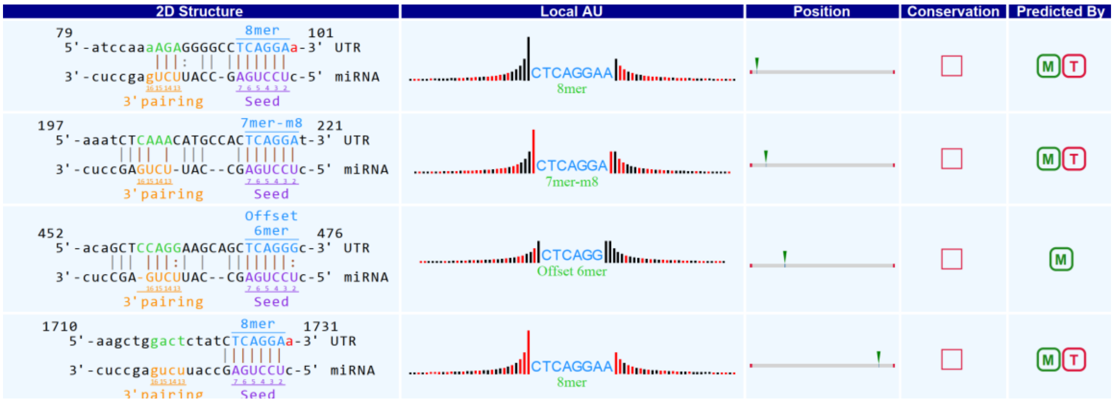

hsa-miR-5586-5p_vs_hsa_circRNA_004658

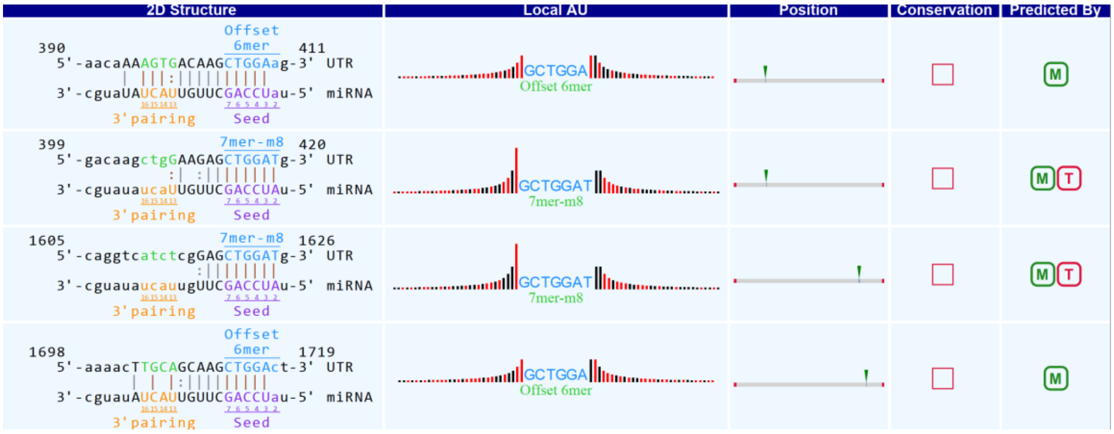

hsa-miR-6762-3p_vs_hsa_circRNA_004658

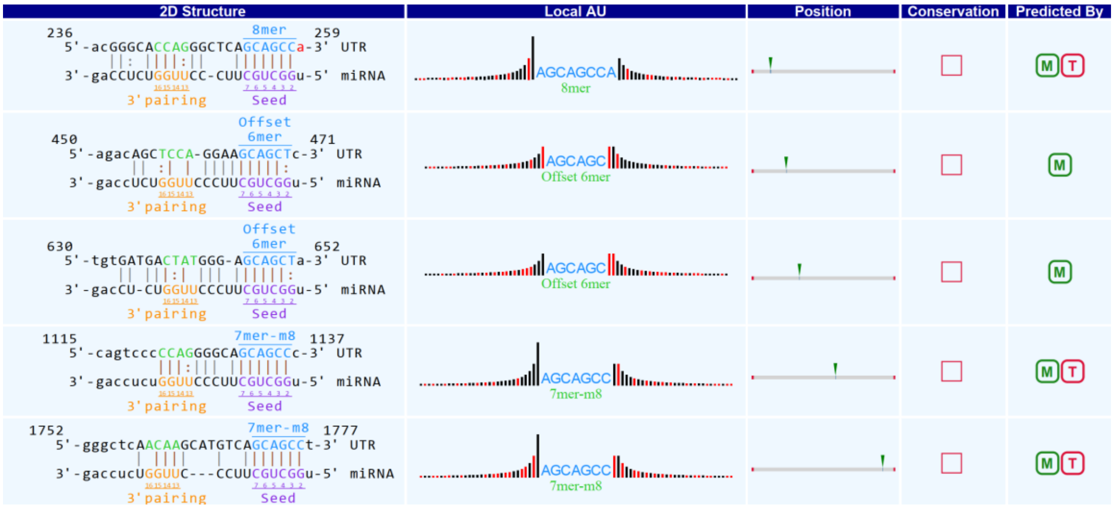

hsa-miR-27a-3p_vs_hsa_circRNA_005232

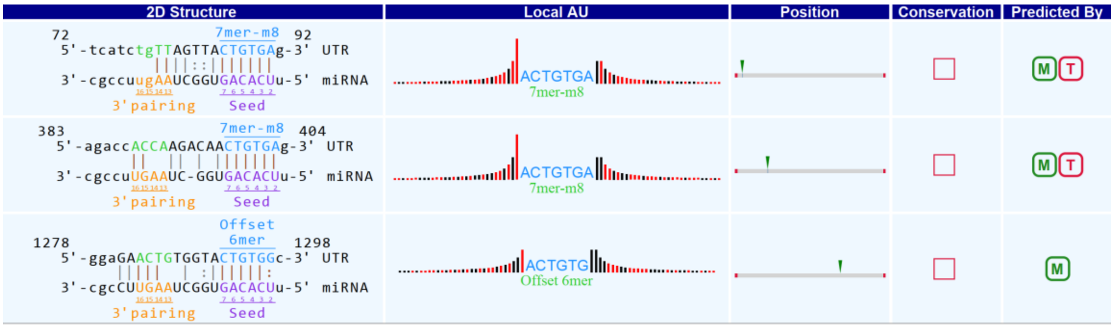

hsa-miR-27b-3p_vs_hsa_circRNA_005232

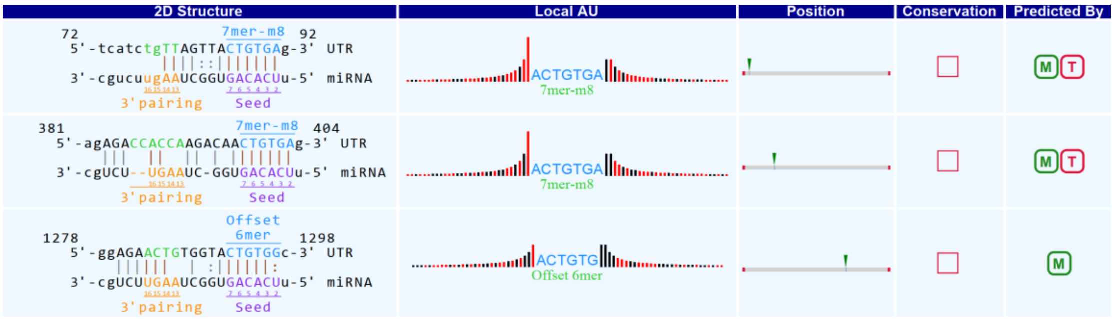

hsa-miR-373-5p_vs_hsa_circRNA_005232

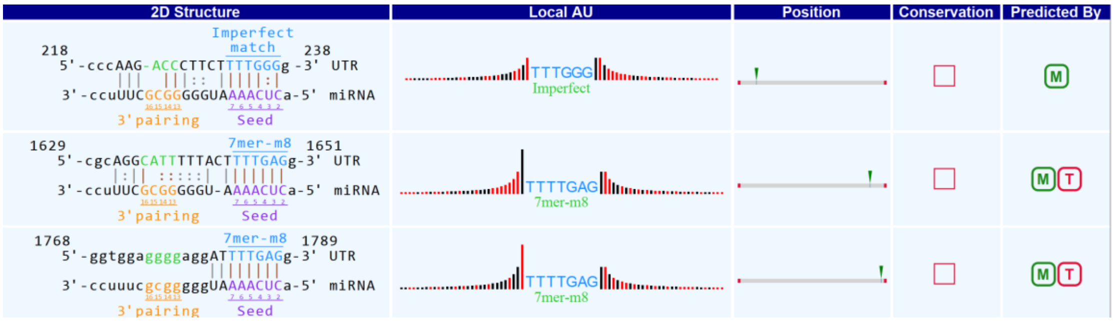

hsa-miR-3180-5p_vs_hsa_circRNA_005232

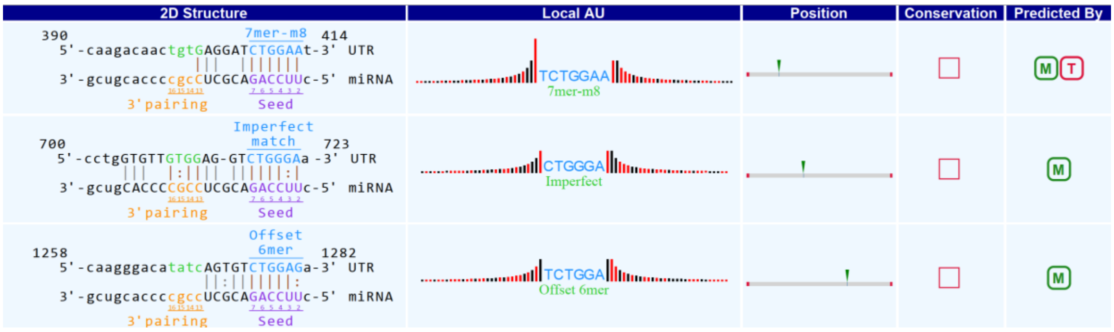

hsa-miR-6888-5p_vs_hsa_circRNA_005232

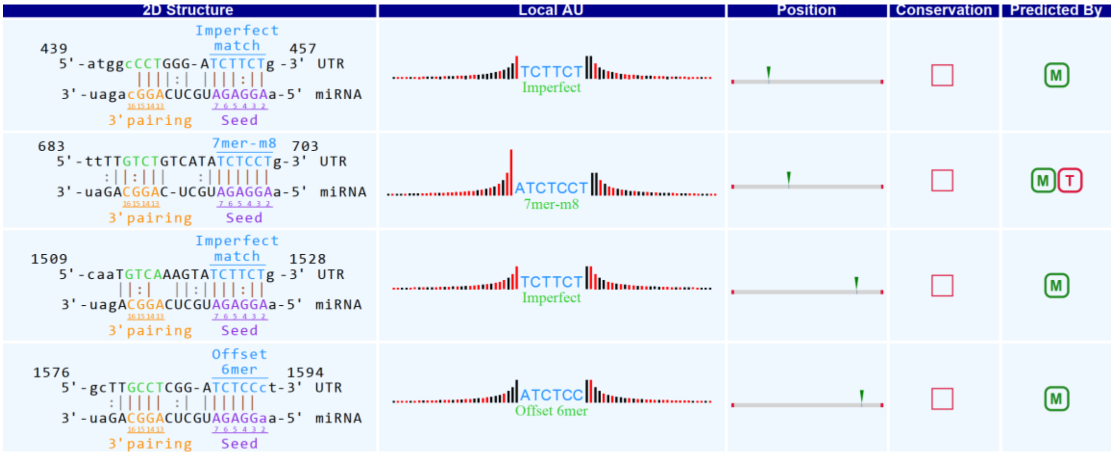

hsa-miR-105-5p_vs_hsa_circRNA_006919

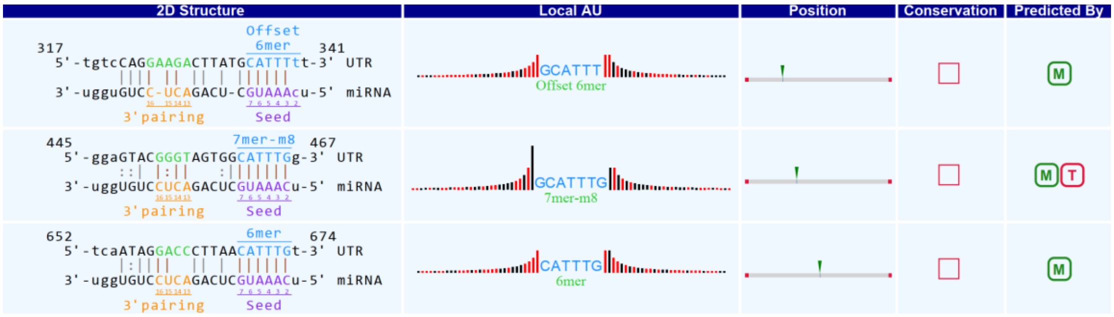

hsa-miR-487a-3p_vs_hsa_circRNA_006919

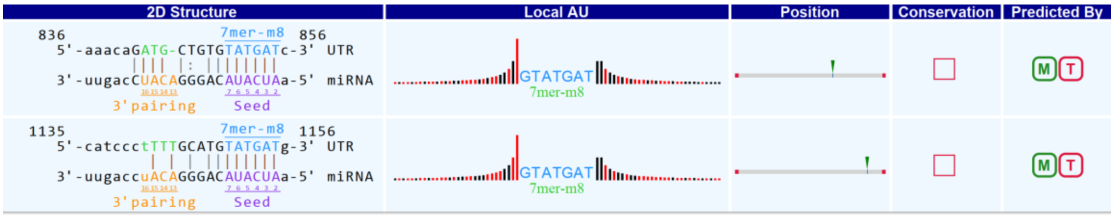

hsa-miR-660-3p_vs_hsa_circRNA_006919

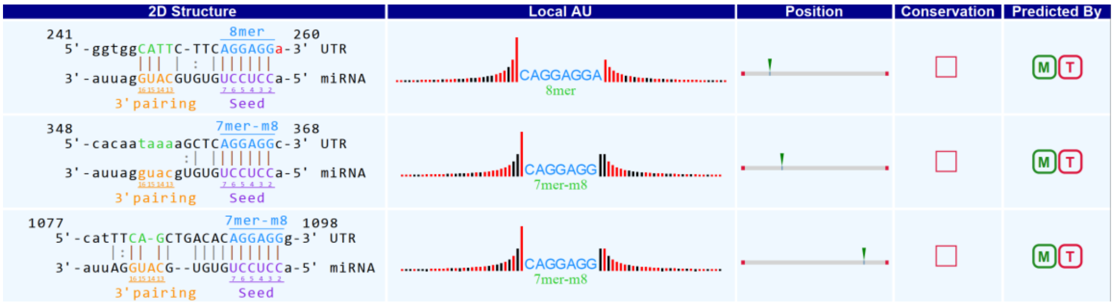

hsa-miR-1226-3p_vs_hsa_circRNA_006919

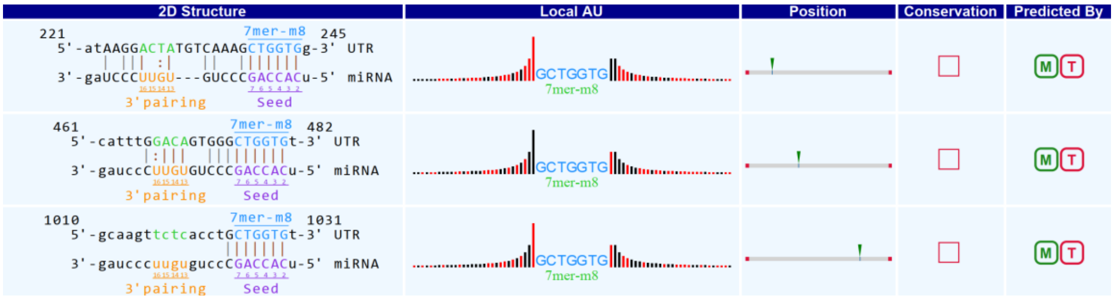

hsa-miR-7152-5p_vs_hsa_circRNA_006919

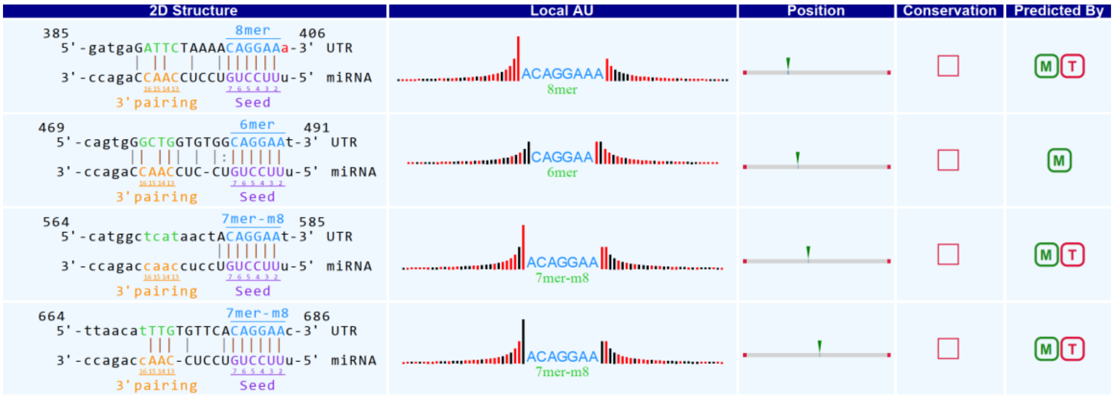

hsa-miR-27a-3p_vs_hsa_circRNA_075671

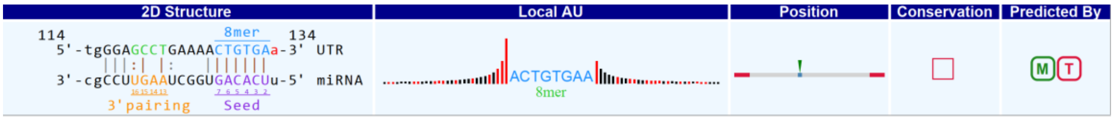

hsa-miR-2682-3p_vs_hsa_circRNA_075671

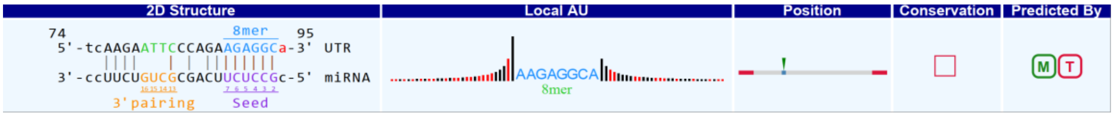

hsa-miR-4778-3p_vs_hsa_circRNA_075671

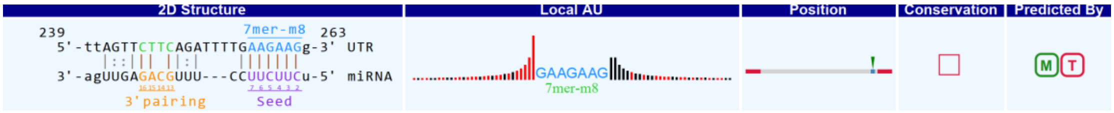

hsa-miR-6882-3p_vs_hsa_circRNA_075671

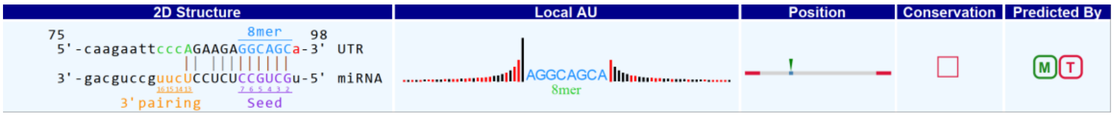

hsa-miR-8083_vs_hsa_circRNA_075671

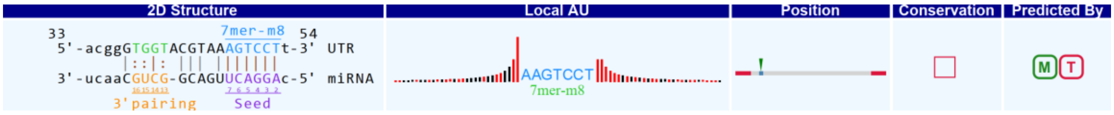

hsa-miR-27a-3p_vs_hsa_circRNA_102700

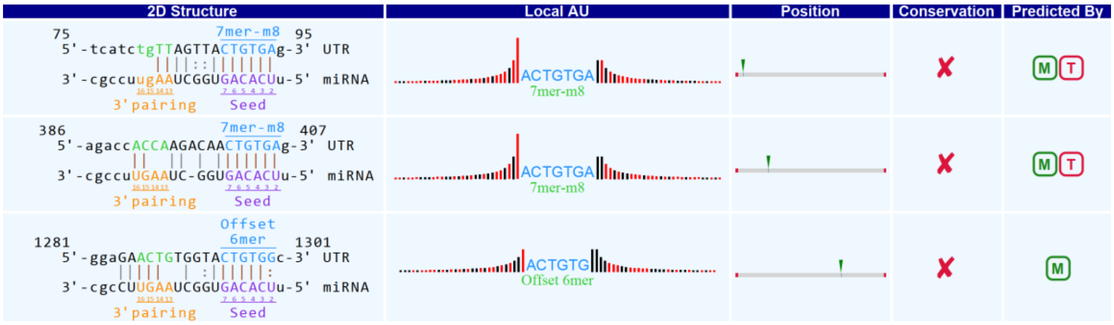

hsa-miR-27b-3p_vs_hsa_circRNA_102700

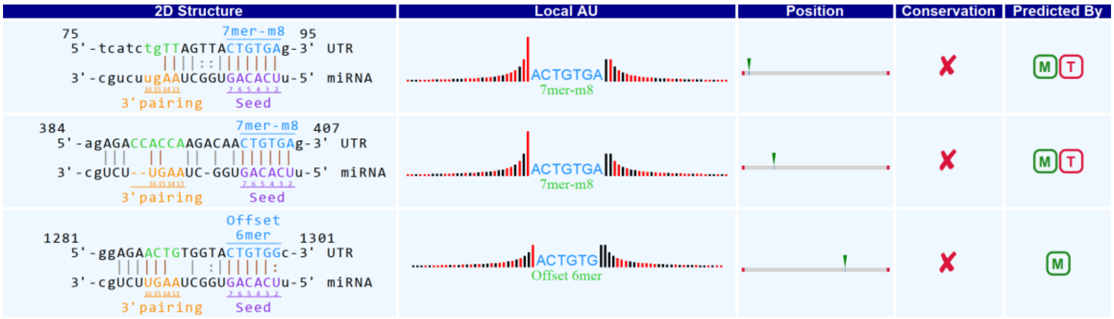

hsa-miR-335-3p_vs_hsa_circRNA_102700

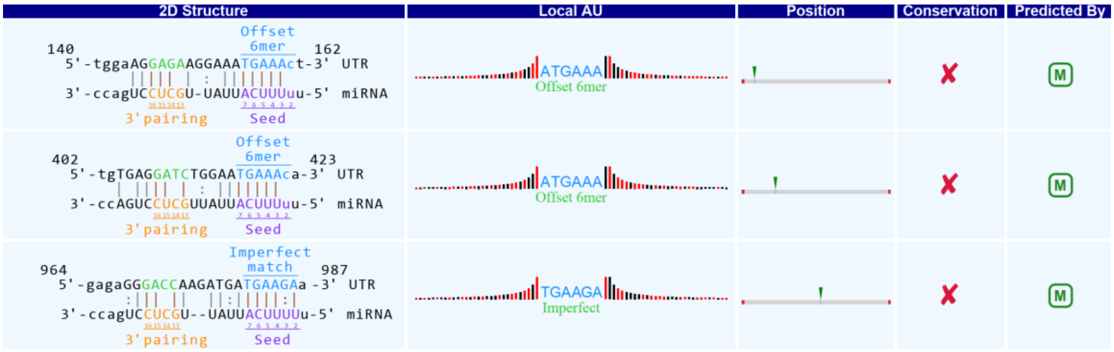

hsa-miR-373-5p_vs_hsa_circRNA_102700

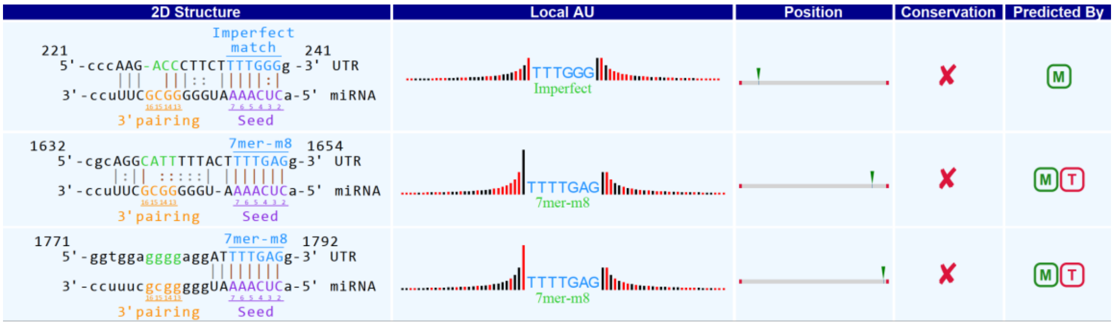

hsa-miR-628-5p_vs_hsa_circRNA_102700

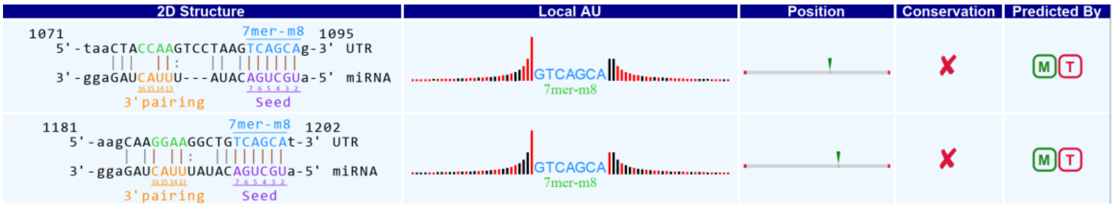

hsa-miR-29b-1-5p_vs_hsa_circRNA_104101

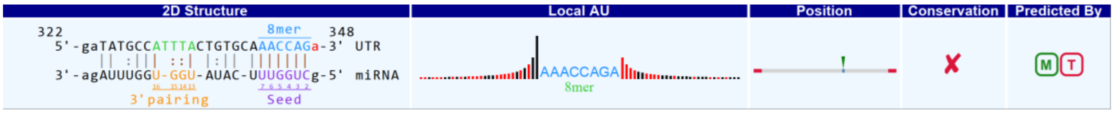

hsa-miR-561-5p_vs_hsa_circRNA_104101

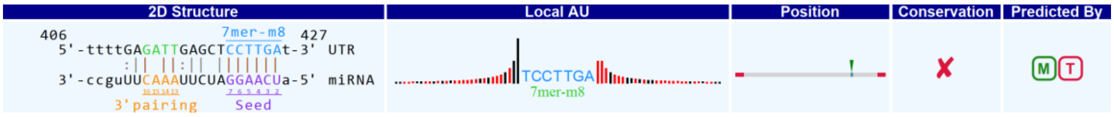

hsa-miR-642a-5p_vs_hsa_circRNA_104101

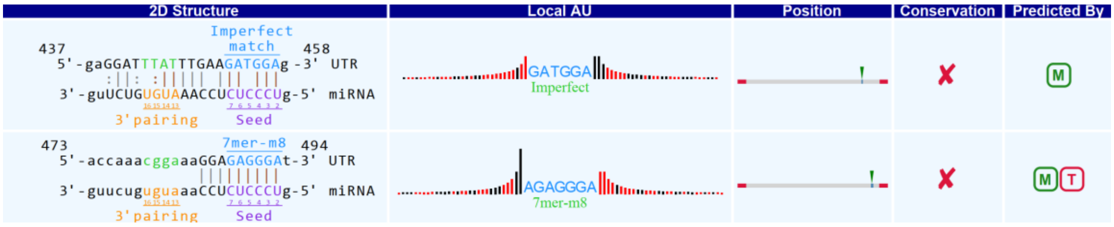

hsa-miR-708-5p_vs_hsa_circRNA_104101

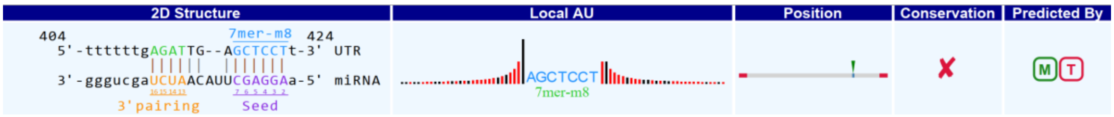

hsa-miR-1468-5p_vs_hsa_circRNA_104101

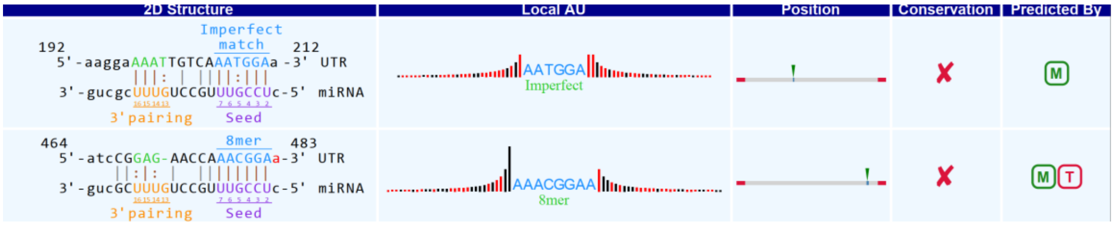

hsa-miR-92a-2-5p_vs_hsa_circRNA_104730

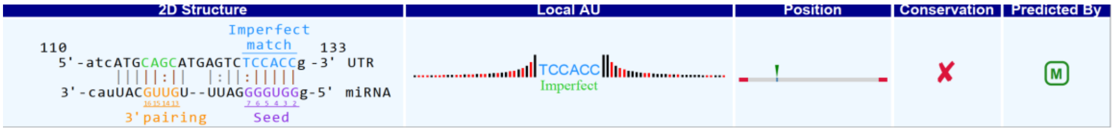

hsa-miR-504-5p_vs_hsa_circRNA_104730

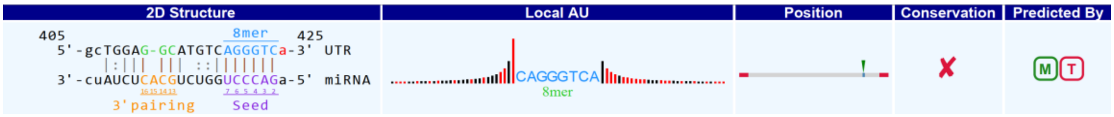

hsa-miR-615-5p_vs_hsa_circRNA_104730

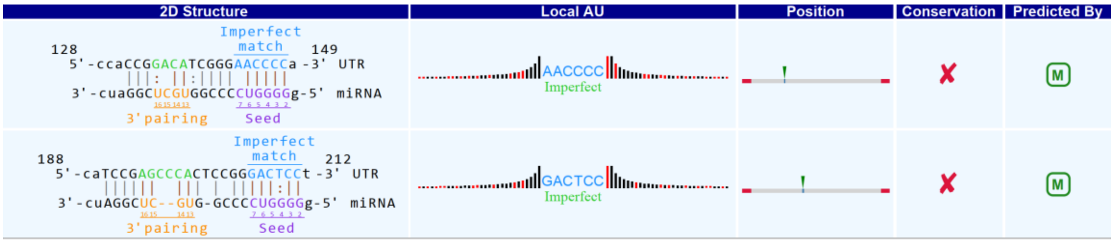

hsa-miR-660-3p_vs_hsa_circRNA_104730

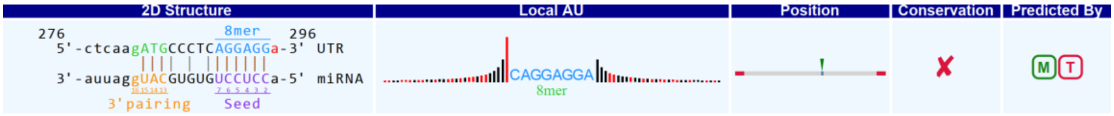

hsa-miR-661_vs_hsa_circRNA_104730

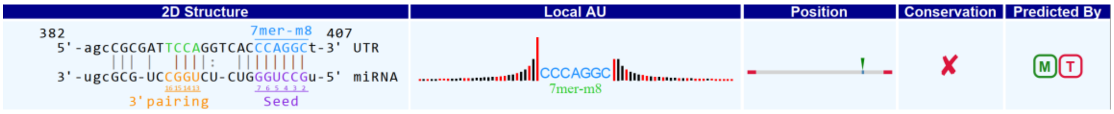

hsa-miR-216a-3p_vs_hsa_circRNA_104981

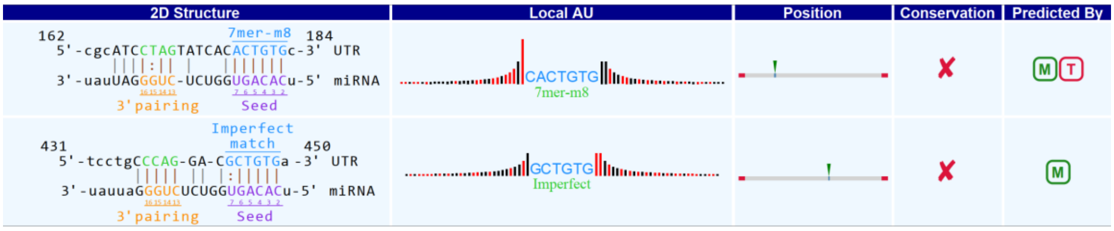

hsa-miR-597-3p_vs_hsa_circRNA_104981

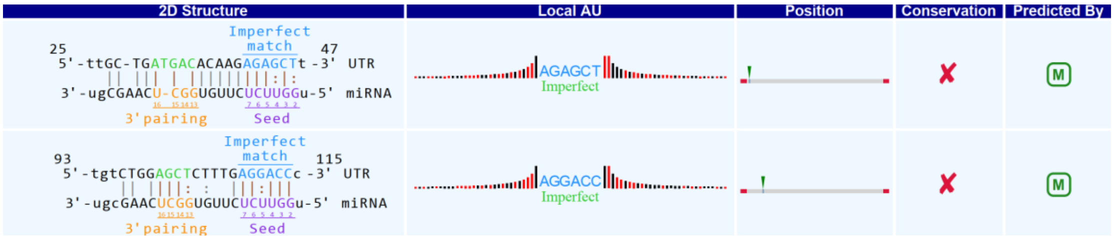

hsa-miR-612_vs_hsa_circRNA_104981

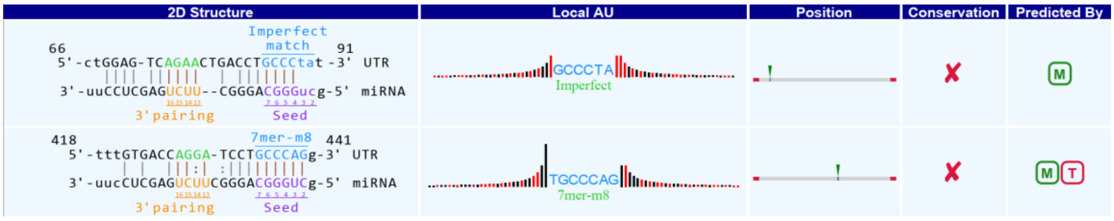

hsa-miR-888-5p_vs_hsa_circRNA_104981

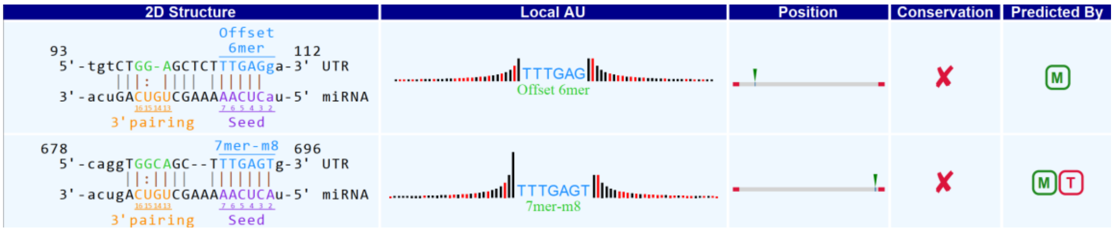

hsa-miR-891a-3p_vs_hsa_circRNA_104981

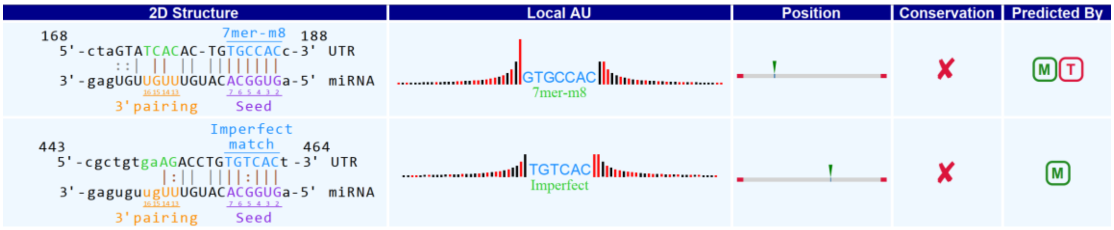

Supplement: Supplementary file 3 — Additional file 3. Appendix 3: CircRNAs target-miRNAs prediction by using TargetScan and miRanda. [file 13287_2022_3160_MOESM3_ESM.pdf]
