# Supplementary figures and images for: Integrative network analysis of circular RNAs reveals regulatory mechanisms for hepatic specification of human iPSC-derived endoderm
Source: Stem Cell Res Ther. 2022 Sep 8;13:468. doi: 10.1186/s13287-022-03160-z (PMC9461288; doi:10.1186/s13287-022-03160-z)

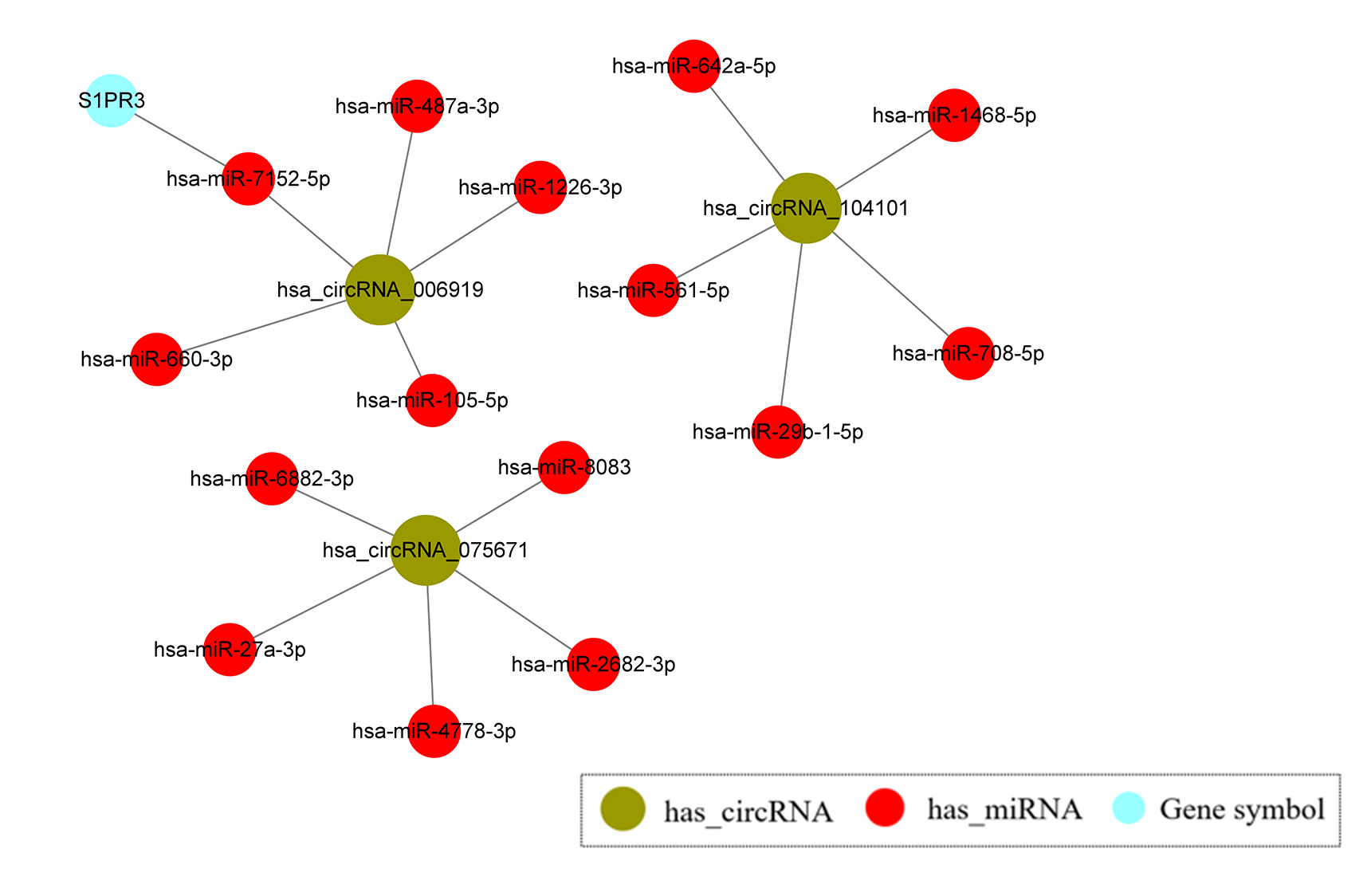

Supplement: Supplementary file 4 — Additional file 4. Appendix 4. Figure S1: CircRNA–miRNA–mRNA regulatory network during hepatic specification. The network of downregulated DEcircRNAs, miRNAs and targeted mRNAs are represented with different shapes (green circle represents hsa_circRNA, red circle represents hsa_miRNA; blue circle represents targeted mRNA). [file 13287_2022_3160_MOESM4_ESM.tif]

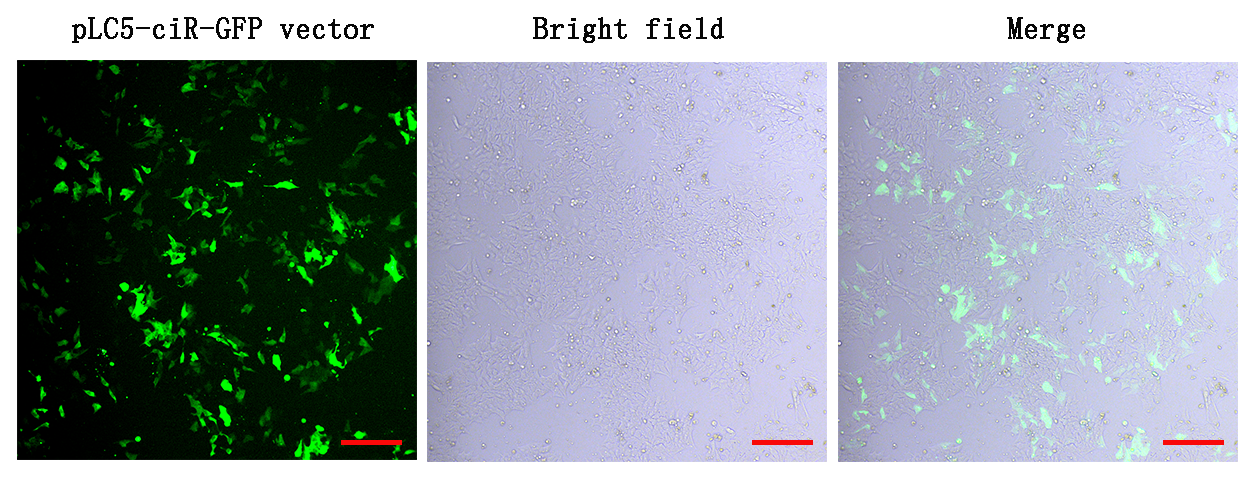

Supplement: Supplementary file 6 — Additional file 6. Appendix 6. Figure S3: Transfection efficiency of DE cells after transinfected with pLC5-ciR-hsa_circ_004658 (GFP) plasmid. [file 13287_2022_3160_MOESM6_ESM.tif]

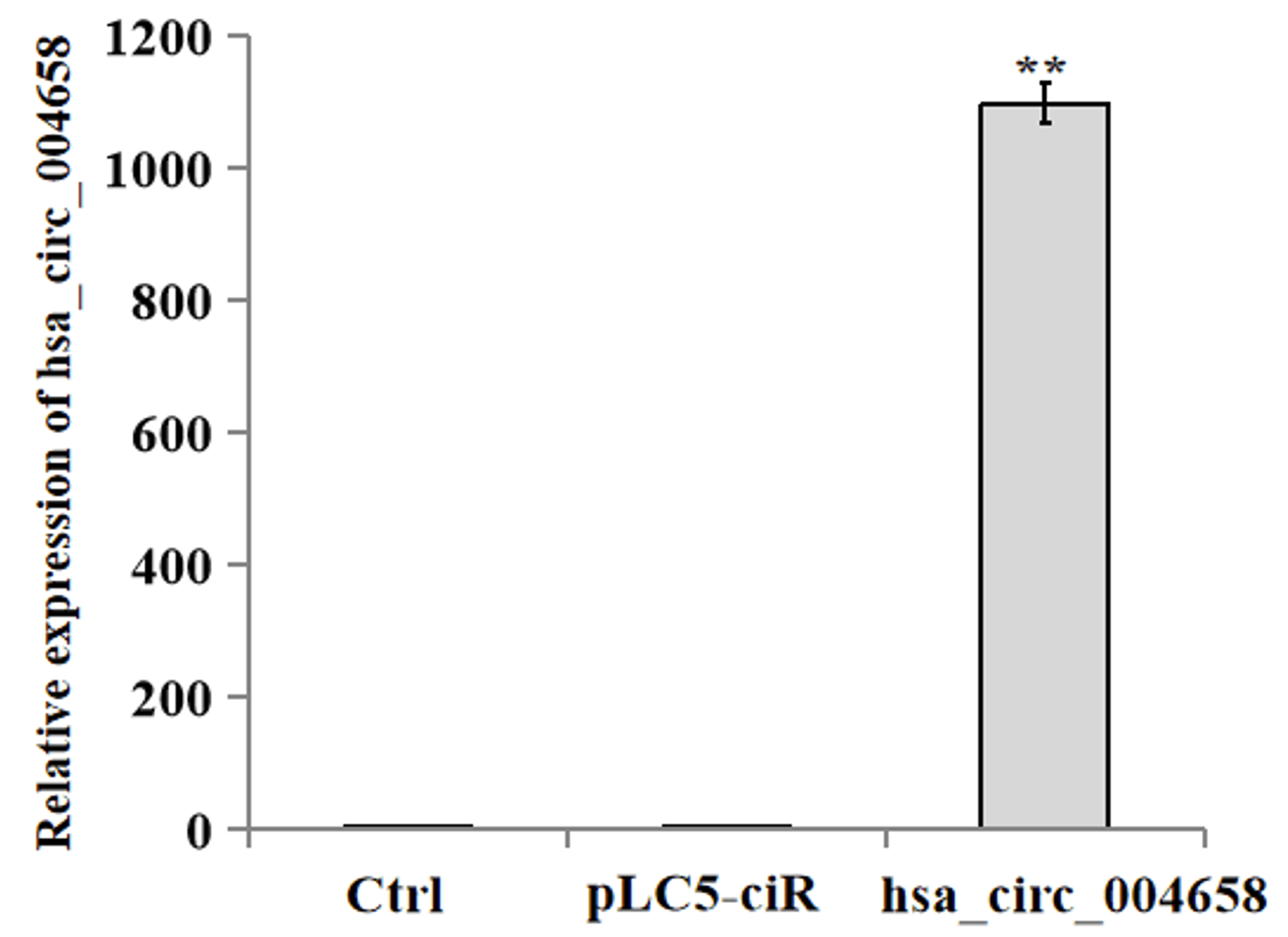

Supplement: Supplementary file 7 — Additional file 7. Appendix 7. Figure S4: Relative expression of hsa_circ_004658 in DE cells after transinfected with pLC5-ciR-hsa_circ_004658. [file 13287_2022_3160_MOESM7_ESM.tif]

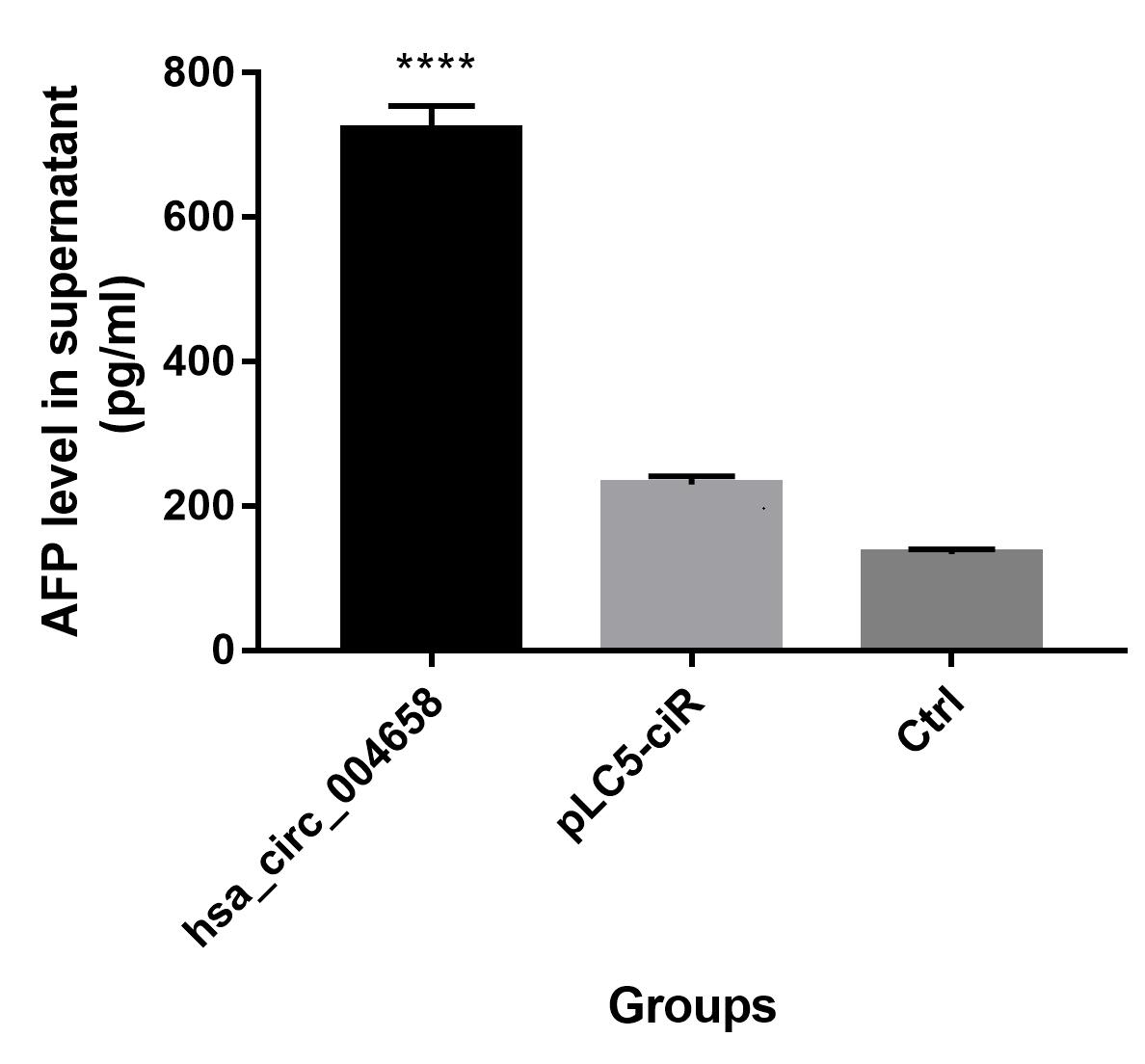

Supplement: Supplementary file 8 — Additional file 8. Appendix 8. Figure S5: The level of AFP in cell supernatant at 54 hours after trans-infection. [file 13287_2022_3160_MOESM8_ESM.jpg]

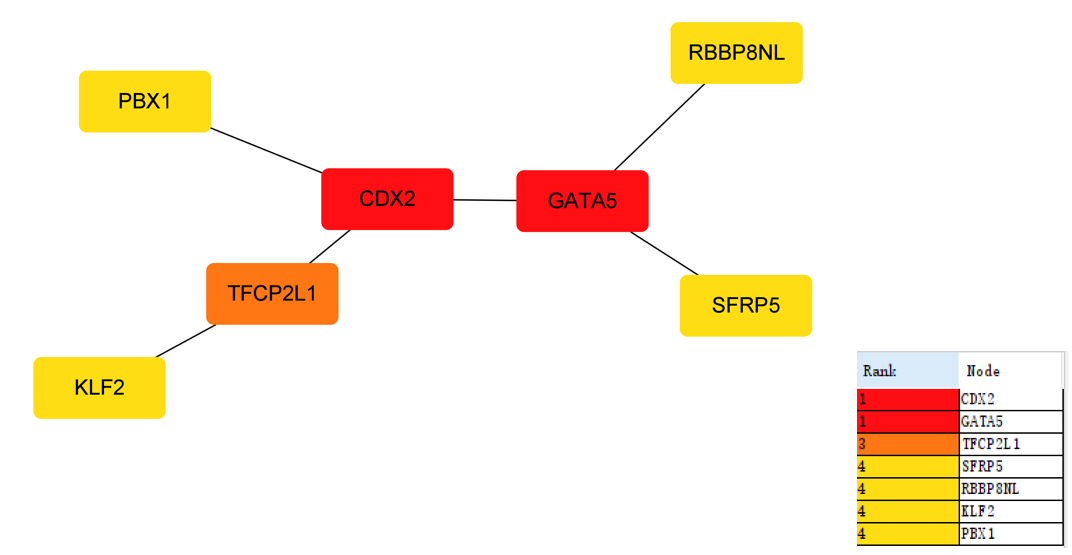

Supplement: Supplementary file 9 — Additional file 9. Appendix 9. Figure S6: Protein-protein interaction network construction and hub genes screening among upregulated DEGs. [file 13287_2022_3160_MOESM9_ESM.png]

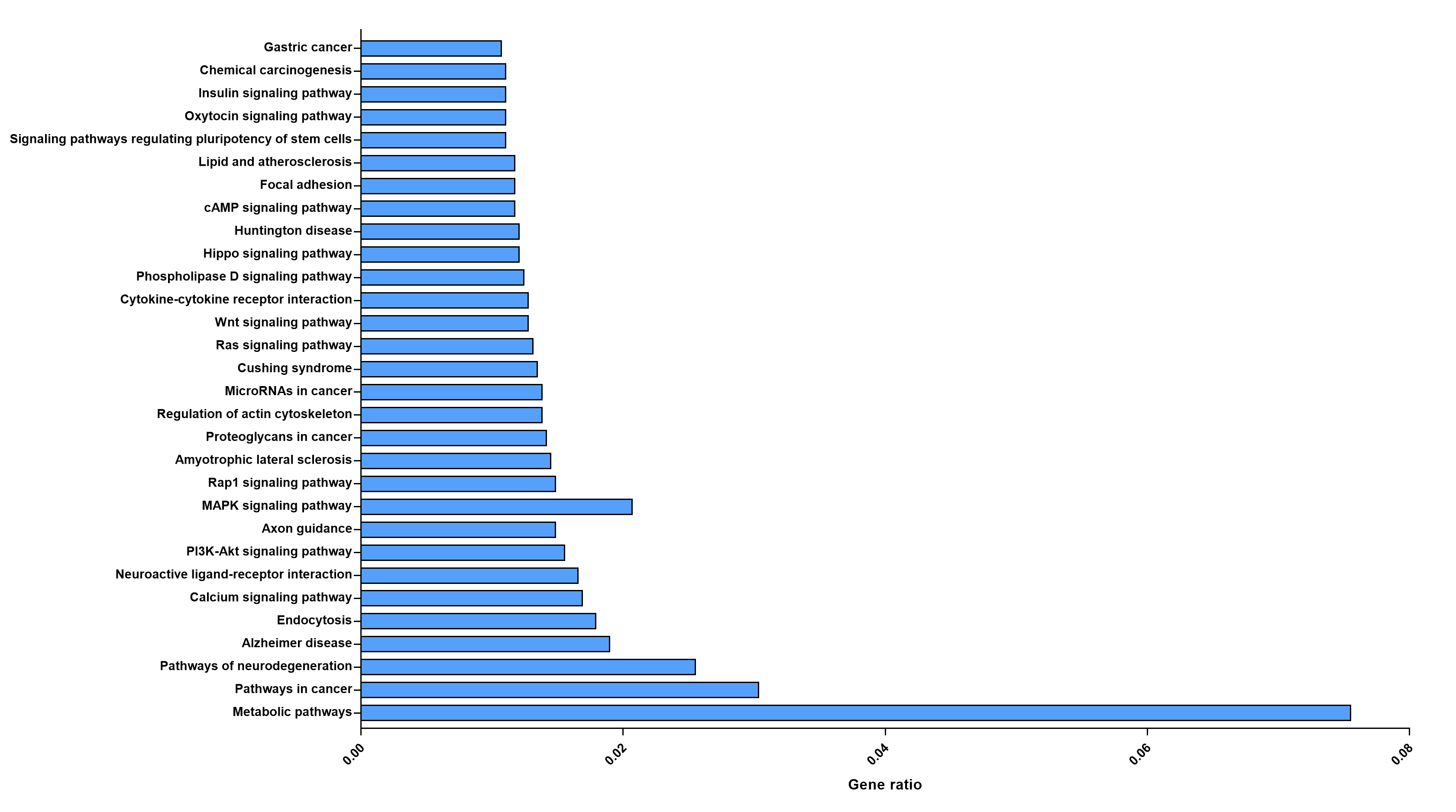

Supplement: Supplementary file 10 — Additional file 10. Appendix 10. Figure S7: KEGG enrichment analysis of 2970 target-genes predicted by hsa_circ_004658. [file 13287_2022_3160_MOESM10_ESM.tif]
