# Supplementary material for: Integrative network analysis of circular RNAs reveals regulatory mechanisms for hepatic specification of human iPSC-derived endoderm
Source: Stem Cell Res Ther. 2022 Sep 8;13:468. doi: 10.1186/s13287-022-03160-z (PMC9461288; doi:10.1186/s13287-022-03160-z)
Supplement: Supplementary file 5 — Additional file 5. Appendix 5. Figure S2: Diagram of the constructed vector pLC5-ciR-hsa_circ_004658. [file 13287_2022_3160_MOESM5_ESM.docx]

C4658-UnF: CATTAATATTTCTTCTTTCGAATTCTAATACTTTCAGATAATGAACCCAGCCAATTCT

C4658-UnR: AGTATGGAGTTGTTAGCTAGGATCCAGTTGTTCTTACTTGCTGATGGCTGAAATTTGA


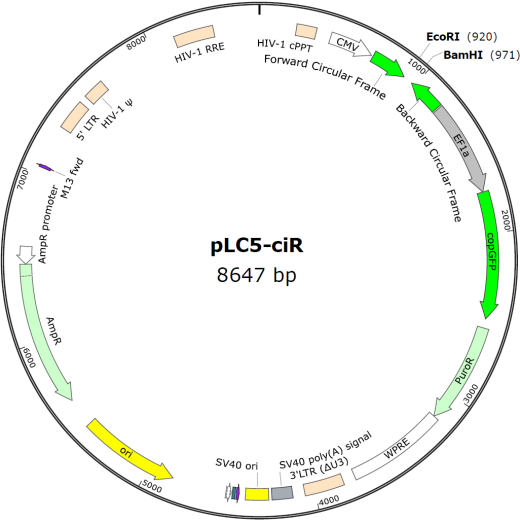


Figure 1 Diagram of pLC5-ciR vector

>hsa_circ_0004658|NM_032048|EMILIN2

ATAATGAACCCAGCCAATTCTCAGAGCCCAGGAAGACTTTGTCCCCAACTGGTACAGCACAACCAAGCTGGGGGGTAGATCCAAAAGAGGGGCCTCAGGAACTTCAGGAAAAGAAGATACAGGTGCTAGAGGAGAAGGTTCTTCGACTCACAAGGACGGTTCTTGACCTCCAGTCTTCCCTTGCTGGAGTGAGTGAAAATCTCAAACATGCCACTCAGGATGATGCCAGTAGAACACGGGCACCAGGGCTCAGCAGCCAGCACCCCAAGCCTGACACCACTGTTAGTGGAGACACAGAAACGGGCCAGAGTCCTGGTGTCTTCAACACTAAGGAATCTGGCATGAAGGACATCAAGTCTGAATTGGCTGAAGTCAAAGATACTCTAAAGAACAAAAGTGACAAGCTGGAAGAGCTGGATGGAAAAGTGAAGGGCTACGAAGGGCAGCTCAGACAGCTCCAGGAAGCAGCTCAGGGCCCGACGGTGACCATGACAACCAACGAACTCTACCAAGCCTATGTGGACAGTAAGATCGACGCCCTGAGAGAGGAGCTCATGGAGGGCATGGACAGAAAGCTGGCTGACCTGAAAAACTCATGTGAGTACAAGCTCACTGGCCTCCAGCAGCAGTGTGATGACTATGGGAGCAGCTACCTGGGAGTGATAGAGCTCATAGGGGAGAAGGAAACAAGCCTGAGAAAAGAAATAAATAACCTCCGAGCCCGGCTACAGGAGCCTTCAGCCCAGGCAAATTGCTGCGACAGTGAAAAGAATGGTGACATTGGTCAACAGATCAAGACATTGGACCAGAAAATCGAGAGAGTTGCTGAAGCCACCAGAATGCTGAATGGAAGACTGGACAATGAGTTTGACCGCCTTATAGTTCCAGAGCCAGATGTGGATTTTGATGCAAAATGGAATGAACTCGATGCAAGGATCAATGTGACGGAGAAGAACGCTGAAGAACATTGCTTTTACATTGAGGAAACCCTTCGGGGCGCCATTAATGGAGAGGTGGGTGACTTGAAGCAGCTTGTTGATCAGAAAATACAGTCTCTGGAAGACCGTCTGGGGAGCGTTCTCCTACAGATGACCAATAACACTGGTGCAGAGCTCAGTCCCCCAGGGGCAGCAGCCCTGCCAGGAGTGTCAGGGTCAGGAGATGAACGGGTCATGATGGAATTAAACCACCTGAAGGACAAAGTTCAAGTTGTTGAAGACATTTGCCTGCTGAACATCCAGGGAAAGCCTCATGGGATGGAAGGTGCCTTGCCAAACAGGGAAGACCGCGCAGTACGCGACAGCCTGCACCTTTTGAAATCTCTCAACGACACGATGCACAGGAAGTTTCAAGAAACCGAACAAACCATCCAGAAACTTCAACAGGATTTTAGTTTTCTTTATTCTCAATTAAACCACACAGAAAATGATGTGACTCATCTTCAAAAGGAAATGAGCAATTGTAGAGCAGGTGAAAACGCTGGCATGGGTAGGTTCACTAAGGTGGGTGAGCAAGAAAGGACAGTGGACACCCTGCCGTCCCCCCAGCACCCCGTGGCTCATTGCTGCAGTCAGCTGGAGGAGAGGTGGCAGAGGTTGCAGAGCCAGGTCATCTCGGAGCTGGATGCTTGTAAGGAATGCACGCAGGGGGTCCAGAGGGAGGTCTCCATGGTGGAGGGCAGGGTGTCTCATATGGAGAAAACTTGCAGCAAGCTGGACTCTATCTCAGGAAATCTTCAGAGGATCAAGGAGGGGCTCAACAAGCATGTCAGCAGCCTGTGGAACTGTGTCAGGCAGATGAACGGAACGCTCAGGTCGCATTCCAGAGACATTTCTGGCCTGAAGAATTCAGTCCAGCAGTTCTACAGCCACGTCTTCCAGATTTCTACTGATTTGCAAGATCTGGTCAAATTTCAGCCATCAGCAA
